# Molecular mechanisms and clinical impacts of sex differences in urologic cancers

**DOI:** 10.1007/s12672-026-05029-6

**Published:** 2026-05-03

**Authors:** Pengcheng Fu, Dong Wen, Geliu Shuai, Yuebin Wang, Shengxin Yu, Yuanri Xie, Pufang Xue, Zhenqin Liang, Junrong Zou, Xiaofeng Zou

**Affiliations:** 1 The First Clinical College, Gannan Medical University, Ganzhou, Jiangxi China; 2 Department of Urology, The First Affiliated Hospital of Gannan Medical University, Ganzhou, 341000 Jiangxi China; 3 Institute of Urology, The First Affiliated Hospital of Gannan Medical University, Ganzhou, 341000 Jiangxi China; 4 Institute of Hematological Research, Shaanxi Provincial People’s Hospital, 256 West Youyi Road, Xi’an, 71000 Shanxi China; 5 Ganzhou Key Laboratory of Rare Earth Materials and Human Health, Ganzhou, 341000 Jiangxi China

**Keywords:** Urologic neoplasms, Sex bias, Sex hormone, Bladder cancer, Androgen receptors

## Abstract

Urological cancers exhibit significant sex differences in incidence, treatment response, and prognosis, with males generally showing higher morbidity and mortality. This review systematically summarizes the underlying molecular and clinical mechanisms of these disparities, focusing on sex hormones, chromosome biology, tumor immune microenvironment, and microbiota. Sex hormones modulate key tumor processes including proliferation, apoptosis, non-apoptotic cell death, and DNA repair. Genetic factors such as X chromosome inactivation escape genes and Y chromosome loss also contribute to sex-biased cancer susceptibility. Furthermore, sex-specific differences in the urinary system and gut microbiota influence local immunity and inflammation, thereby affecting tumor progression and therapeutic response. Lifestyle and environmental factors, including smoking, alcohol consumption, and occupational exposures, further exacerbate these disparities. Clinically, sex differences impact the efficacy of immunotherapy and targeted therapies, underscoring the need for sex-informed treatment strategies. Integrating sex as a biological variable in research, clinical practice, and public health policies is essential for advancing precision oncology in urologic cancers.

**Supplementary Information:**

The online version contains supplementary material available at 10.1007/s12672-026-05029-6.

## Introduction

**Fig. 1:**
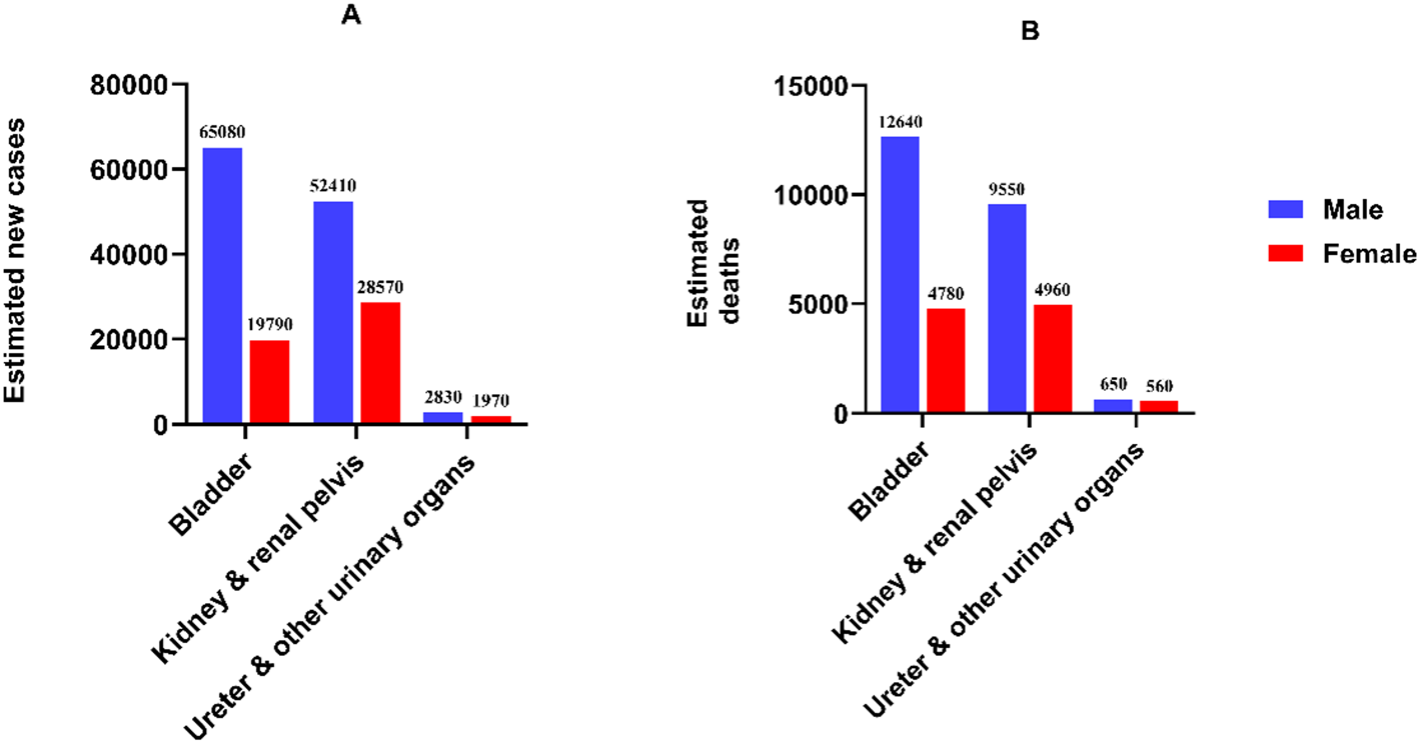
Gender disparities in the estimated number of new cases and deaths for urinary system cancers in the United States, 2025. **A** Estimated new cases. **B** Estimated deaths. Data are presented for bladder cancer, kidney & renal pelvis cancer, and ureter & other urinary organs cancer Data source: [2]

**Fig. 2:**
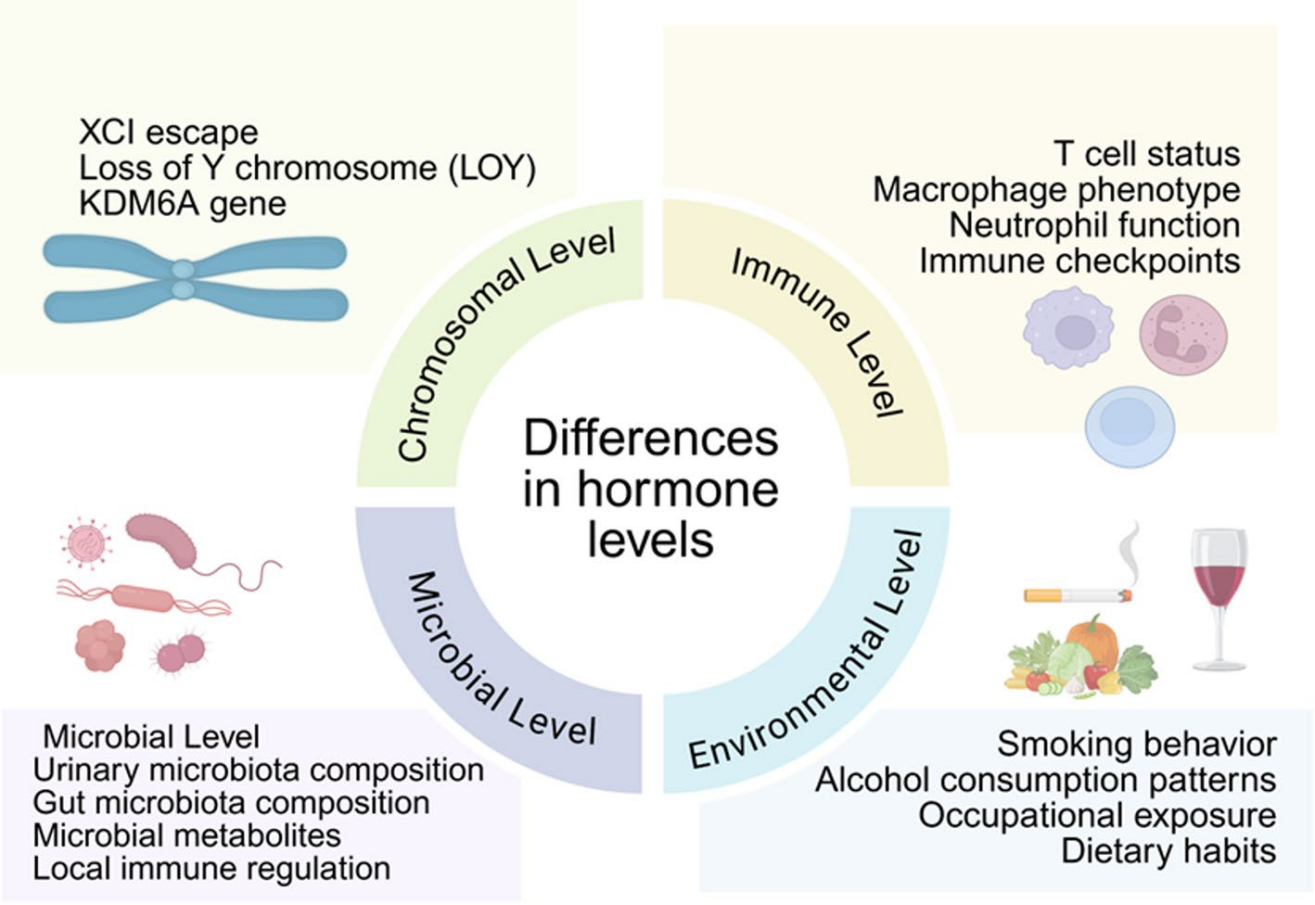
Multidimensional mechanisms underlying sex differences in tumor biology.The schematic illustrates the four interconnected dimensions contributing to sex disparities in cancer: chromosomal/epigenetic factors (XCI escape, LOY, KDM6A), immune/hormonal regulation (T cell status, macrophage polarization), microbial/local environment (microbiota composition), and lifestyle/environmental exposures (smoking, diet, occupation). Central to these interactions are intrinsic differences in sex hormone levels

Urologic cancers exhibit significant sex disparities, with men showing higher incidence and mortality in malignancies such as bladder and kidney cancer [1, 2] (Fig. 1). These differences are influenced not only by lifestyle and environmental factors but also by intrinsic biological mechanisms. This review aims to synthesize current evidence on the biological basis of sex differences in urologic cancers, focusing on sex hormones [3], chromosome effects [4], immune microenvironment [5], and microbiota [6]. A systematic literature search was conducted using PubMed, Web of Science, and Scopus, with keywords including “urologic neoplasms,” “sex bias,” “sex hormones,” and “immunotherapy.” Evidence indicates that sex hormones and their receptors regulate key tumor processes including proliferation [7], apoptosis [8], and DNA repair [9]. Sex chromosome genes, especially those escaping X-inactivation [4] and Y chromosome genes [10], also contribute to cancer risk. Additionally, sex-specific differences in urinary [11] and gut [6] microbiota influence local immunity and inflammation, further modulating cancer development and treatment response. Despite growing knowledge, the integration of sex-specific factors into research and clinical practice remains inadequate. This gap has direct clinical consequences. Understanding sex differences is critical for optimizing treatment selection predicting patient response, developing sex-specific biomarkers, and designing balanced clinical trials. This review highlights these mechanisms and discusses their implications for developing sex-informed therapeutic strategies, ultimately supporting the advancement of precision medicine in urologic oncology (Fig. 2).

## Hormonal mechanisms in urologic cancers

### Regulation of physiological processes by sex hormones

#### Sex hormones and cell proliferation

**Fig. 3:**
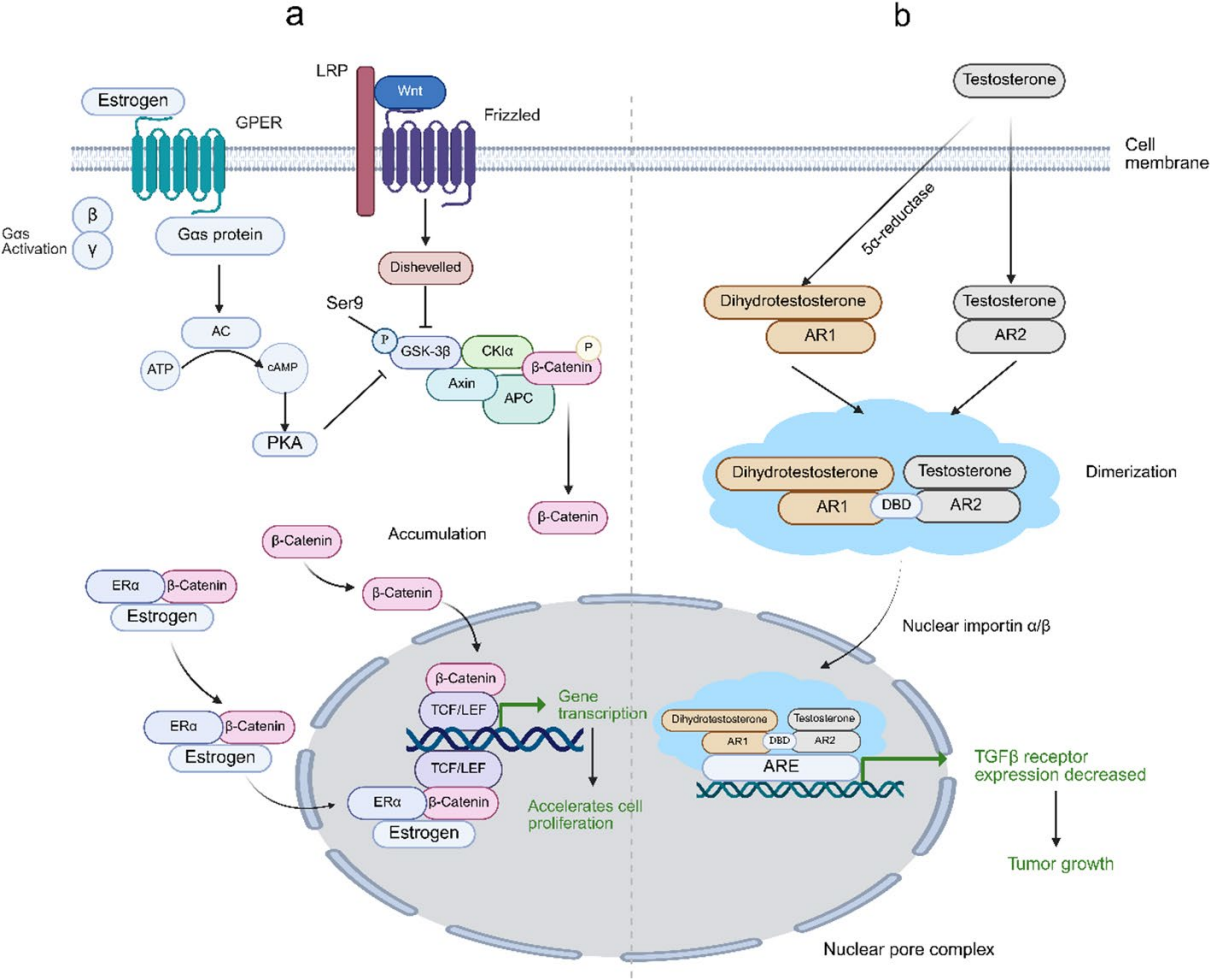
**a** Estrogen-binding plasma membrane G protein-coupled estrogen receptor (GPER) activates Gs protein, induces adenylyl cyclase (AC) to catalyze ATP to produce cAMP, and activates protein kinase A (PKA). PKA phosphorylates glycogen synthase kinase 3β (GSK-3β) serine (p-Ser9) at position 9, inhibits its kinase activity, blocks the function of β-catenin (β-catenin) degradation complex, and leads to the accumulation of phosphorylated β-catenin in the cytosol. After translocation of β-catenin into the nucleus, it forms a transcriptional complex with T cytokine/lymphoid enhancer (TCF/LEF). It up-regulates the expression of cyclin D1 to drive cell proliferation. At the same time, estrogen diffuses into the cell. It binds to the nuclear receptor ERα, which directly enhances the transcriptional activity of the β-catenin/TCF/LEF complex and synergistically promotes the expression of Wnt target genes. **b** After testosterone crosses the plasma membrane, it is partially converted into dihydrotestosterone (DHT) catalyzed by 5α-reductase, which binds to the androgen receptor (AR) to form a hormone-AR complex, respectively. The two monomers are dimerized by a DNA-binding domain (DBD) and transported into the nucleus via the nuclear pore complex (NPC) mediated by the Importin α/β complex. Intranuclear AR dimers specifically bind to androgen response elements (AREs) and inhibit the transcription of transforming growth factor β receptor (TGFβR) genes, resulting in down-regulation of TGFβ receptor expression, relieving its tumor suppressor function, and indirectly promoting tumor progression. This figure is an original schematic created by the authors to summarize key mechanisms based on the cited references.

Sex hormones regulate cell proliferation by binding to nuclear receptor or by influencing other signaling pathways. In the pro-cancer mechanism, estrogen induces the expression of Cyclin D1 by activating the Wnt/β-catenin pathway [12], accelerates the G1/S phase transition, and promotes cells to enter the S phase (Fig. 3) [7]. However, its effects are tissue-specific; in uterine leiomyomas, estrogen enhances vitamin D3 synthesis and activation, which inhibits proliferation via vitamin D receptor signaling [13–15].

Androgens downregulate TGF-β receptor I (TβRI) expression through Androgen receptor(AR), attenuating the tumor suppressive function of TGF-β (Fig. 3) [16–18]. After the inactivation of TGF-β signaling, its original proliferative inhibition and pro-apoptotic effects are relieved, and the migration and invasion of tumor cells are promoted instead [18]. Androgen inhibition of the TGF-β signaling pathway may also further promote tumor initiation and progression by affecting immune cell function in the tumor microenvironment [19]. Dihydrotestosterone (DHT) can also promote BCa proliferation and invasion via a non-AR-dependent pathway by binding to Epiplakin1 (EPPK1), which activates the p38 MAPK/c-Jun axis and upregulates Junction Plakoglobin (JUP) expression [20]. Clinical data have shown that high JUP expression is significantly associated with poor prognosis in patients, and blocking this pathway (e.g., knockdown of EPPK1) is effective in slowing tumor progression [20, 21]. This mechanism reveals the critical role of the EPPK1-mediated MAPK/JUP signaling axis in BCa [22], providing a new strategy for targeted therapy.

#### Sex hormones and apoptosis

Sex hormones regulate apoptosis, influencing urological tumor progression. Estrogen can inhibit apoptosis by suppressing MAPK signaling pathway activity [3]. Estrogen can inhibit the expression of Fas and FasL, thereby reducing the occurrence of apoptosis [23]. Estrogen can also influence apoptosis by modulating the activity of key proteins in the death receptor signaling pathway, such as caspase-8 [23].

Androgens enhance the proliferation, invasion, and anti-apoptotic capacity of BCa cells through AR [20]. For example, dihydrotestosterone can promote tumor progression by activating AR signaling, upregulating oncogenesand inhibiting the expression of apoptosis-related proteins [22]. In male-specific prostate cancer, androgen/AR signaling drives tumor progression by activating growth factor pathways, promoting cell proliferation and inhibiting apoptosis in prostate cancer [24, 25], and AR signaling also maintains tumor cell survival by regulating cell cycle-related genes (e.g., p21, p15, p16) and DNA repair mechanisms [26]. The above conclusions suggest that sex hormones play a complex role in a variety of signaling pathways, thereby finely regulating apoptosis.

#### Sex hormones and non-apoptotic cell death

In addition to classical apoptosis regulation, sex hormones are also involved in regulating a variety of non-apoptotic cell death pathways, including ferroptosis [27]、pyroptosis [28], and autophagy [28].These mechanisms contribute to sex differences in urinary cancer development.

Ferroptosis is an iron-dependent form of regulated cell death driven by lipid peroxidation, the occurrence of which is closely associated with cellular metabolic status and levels of oxidative stress [29]. In gynecological cancers, estrogen’s antioxidant properties can suppress ferroptosis by upregulating GPX4, a mechanism that may be relevant in hormone-responsive urologic tumors [29, 30]. A clear interaction exists between AR signaling pathways and ferroptosis [31]. Antiandrogen agents, when combined with iron-containing compounds, can enhance ferroptosis by promoting oxidative damage. Meanwhile, androgen deprivation therapy may influence cellular susceptibility to ferroptosis through alterations in the expression of iron metabolism-related genes [32]. In addition, the PI3K–AKT–mTOR signaling pathway is also involved in the regulation of ferroptosis. In prostate cancer, this pathway is modulated by androgen signaling and can suppress lipid peroxidation via the SREBP1/SCD1 axis, thereby conferring resistance to ferroptosis [33].

Pyroptosis is an inflammatory mode of programmed cell death mediated by gasdermin proteins, characterized by the extensive release of inflammatory factors that significantly influence the immune status of the tumor microenvironment [34, 35]. It has dual roles: inducing anti-tumor immunity or, if excessive, promoting immunosuppression [36]. The inflammation-dependent mechanism of pyroptosis provides a theoretical basis for its potential regulation. Future studies should explore the interaction of sex hormones with these pathways to elucidate their specific roles in tumor immunity.

As a crucial intrinsic self‑regulatory mechanism, the interplay between autophagy and sex hormones in tumorigenesis is spatiotemporally dynamic and tissue‑specific [37]. Estrogen exerts bidirectional regulation via the autophagy pathway: early activation of cytoprotective autophagy serves to restrain tumor initiation, whereas in advanced stages, autophagy is co‑opted to facilitate tumor adaptation and survival [38]. In contrast, androgens predominantly display unidirectional regulatory features that promote tumor progression [39]. Gender differences markedly influence basal autophagic activity as well as tumor susceptibility, and such interactions are intimately linked to clinical therapeutic resistance [40, 41]. Targeting the “sex hormone–autophagy” regulatory axis holds promise as a novel therapeutic strategy for hormone‑dependent tumors exhibiting gender‑related sensitivity.

#### Sex hormones and DNA repair mechanisms

Estrogen signaling is known to modulate DNA repair pathways, influencing therapy resistance in hormone-responsive cancers. In breast cancer models, estrogen via ERα enhances repair pathways like homologous recombination and base excision repair, contributing to radio- and chemoresistance [9, 42, 43]. Whether similar estrogen-driven DNA repair mechanisms operate in urologic cancers like BCa remains largely unexplored and represents a key knowledge gap. Among them, the ER signal activates the Base Excision Repair (BER) pathway, which regulates the activity of repair enzymes such as OGG1 to remove oxidative damage products such as 8-oxygen guanine [44]. Disparities and Trends. However, in the obesity-related microenvironment, estrogen synergizes with leptin, insulin, and other factors to increase the accumulation of DNA damage and form a cancer-promoting environment [45].In epigenetic regulation, the ER recruits epiregulators such as histone demethylase (KDM4B) to remodel chromatin accessibility at sites of DNA damage [46, 47]. This regulation may both enhance the efficiency of repair in breast cancer and may lead to an increase in the rate of repair errors.

In prostate cancer, AR directly upregulates CRY1, a transcriptional coregulator that enhances the efficiency of non-homologous end joining (NHEJ) DNA repair. High CRY1 expression is linked to poorer patient prognosis [48]. AR does not directly regulate the transcription of DNA damage response genes [49], and AR signaling upregulates the expression of DNA damage response genes such as ATM and RAD51, such as in prostate cancer cells carried by the HSD3B1 (1245 C) allele, AR activation leads to enhanced expression of genes related to DNA double-strand break repair, accelerates DNA repair and promotes radiotherapy resistance [50]. This underpins the efficacy of Poly ADP-ribose Polymerase inhibitors in homologous recombination-deficient prostate cancer [51].

### Expression patterns and functional roles of sex hormone receptors in urologic cancers

#### Dual role of the estrogen receptor

Estrogen receptor α (ERα) and β (ERβ) are the two main estrogen receptor subtypes, and they are known to differ significantly in structure and function. In BCa tissues, the mRNA and protein expression levels of ERα were significantly higher than those in normal tissues, especially in immortalized urothelial cells and bladder tumor cells, while the expression levels of ERβ were nearly identical in the two tissues [52]. However, a small number of immunohistochemical studies have found that the expression of ERα in BCa tissues may be reduced compared with non-tumor tissues, suggesting that the expression of ERα may be affected by the tumor microenvironment or detection methods [53]. In general, the effect of ERα may promote tumor proliferation, etc., while the expression of ERβ is not significantly different, and there may be anti-tumor effects.

ERα can also promote cell cycle progression and tumor growth by activating signaling pathways such as MAPK/ERK and PI3K/AKT [3]. ERα expression is upregulated in malignant transformed prostate epithelial cells and increased in malignant and metastatic prostate cancers, and ERα signaling can also promote tumor cell invasion by regulating the degradation of the extracellular matrix and cell motility [54].

ERβ is mainly expressed in epithelial cells, has protective and anti-apoptotic effects, and is able to inhibit the proliferation and migration of tumor cells. ERβ can downregulate the expression of CyclinD1 and prevent cells from moving from G1 phase to S phase, thereby slowing down the rate of cell proliferation, while ERβ can also upregulate some genes that inhibit cell proliferation, such as p21 and p27, to further inhibit cell proliferation [55, 56]. ERβ is cross-regulated with AR, directly or indirectly influencing AR expression and inducing apoptosis and inhibiting tumor progression by competing with AR to bind to or regulating the expression and activity of intracellular transcription factors [57]. It is important to note that different splice variants of ERβ may have different functions, and in breast cancer, splice variants such as ERβ2 and ERβ5 are associated with high Ki-67 positivity, poor prognostic markers, and promotion of cell migration and invasion [58]. Whether this applies to urinary tract tumors requires further investigation. However, the lack of specificity of existing antibodies leads to contradictory results in the study of splice variants, and further experiments are needed to verify their function.

The opposing functions of ERα and ERβ in urologic cancers are largely attributed to their distinct cellular distributions: ERα predominates in stromal/mesenchymal cells, often associating with pro-tumorigenic pathways, while ERβ is mainly expressed in epithelial cells and frequently correlates with anti-proliferative effects [55, 56]. This functional dichotomy, however, is not absolute and can be modulated by factors like androgen levels, which may suppress ERβ activity and tilt the balance toward ERα-driven oncogenesis [59]. Critically, the net effect of estrogen signaling in a given tumor likely depends on the ERα:ERβ expression ratio, receptor splice variants, and crosstalk with other pathways [60, 61]. Targeting this regulatory axis with selective estrogen receptor modulators (SERMs) represents a rational therapeutic strategy to exploit this functional conflict [62].

#### AR expression in bladder cancer progression

AR is sex-significantly expressed in BCa, and AR expression levels are higher in men than in women, which may partly explain the higher prevalence of BCa in men than in women [22]. However, AR expression levels are inversely correlated with tumor stage and grade: AR expression is high in low-grade (Ta-T1) non-muscle-invasive bladder cancer (NMIBC), but significantly decreases in high-grade or muscle-invasive bladder cancer (MIBC) [63, 64]. Changes in the expression of AR dynamics suggest that it may have a pro-tumor effect in the early stages of BCa, but its expression is gradually suppressed as the tumor progresses, which may be associated with tumor dedifferentiation or metastasis [65, 66].

### Immune modulation by sex hormones

#### Neutrophils

Estrogen alters gene expression and functional activity in neutrophils. In breast tumor models, estrogen activates neutrophils to release pro-inflammatory factors (e.g., IL-1β, IL-8) to form a pro-tumor microenvironment conducive to tumor growth [67].

We know that the high incidence of bladder cancer is associated with androgen levels in men Androgens suppress neutrophil cytotoxicity by upregulating TGF-β receptor I (TβRI). This suppression can be reversed by either high-dose testosterone or TβRI inhibitors, restoring anti-tumor activity [19].Programmed cell death ligand 1 (PD-L1) expressed by tumor cells blocks neutrophil cytotoxicity, while androgens may further inhibit its antitumor effects by enhancing PD-L1 expression [19, 68]. AR signaling can also block T cell function by upregulating USP18 by upregulating NF-κB activity [69], indirectly affecting neutrophil function and forming an immunosuppressive tumor microenvironment. Laura Pala et al. showed that androgen deprivation therapy (ADT) enhanced the efficacy of anti-PD-1 (Programmed cell death protein 1) immunotherapy [70], indirectly reflecting the inhibition of T cell function by AR signaling.

#### Macrophage

Classical models divide macrophages into two polarized states: M1 (pro-inflammatory anti-tumor) and M2 (anti-inflammatory pro-tumor) [71]. The M1 macrophages mediate cytotoxicity via mechanisms like ADCC and T/NK cell activation, whereas M2 macrophages promote immunosuppression, angiogenesis, and metastasis through secretion of factors such as IL-10 and Vascular Endothelial Growth Factor (VEGF) [72]. This binary classification is particularly important in the tumor microenvironment, as M2 macrophages are often associated with immune evasion [73].

E2 can modulate the NF-κB signaling pathway by enhancing IκBα levels through ERα and influencing p65 binding at inflammatory gene promoters [73]. Estrogen also signals through the non-canonical receptor G Protein-Coupled Estrogen Receptor 1(GPER1) to inhibit macrophage proliferation and PD-L1 expression, primarily by suppressing the MEK/ERK/cyclin D1 pathway [74]. Estrogen also inhibits IL-1β production by binding to estrogen receptors (ERs) in neutrophils and macrophages, and reduces its pro-inflammatory effects [75]. These mechanisms lead to enhanced anti-inflammatory properties of M2-type macrophages. Barcena et al. noted that estrogen inhibits the pro-inflammatory phenotype of macrophages, namely M1 type, by upregulating Sirt3 expression [76]. In urologic tumors, the enrichment of M2-type macrophages is associated with tumor metastasis and the immunosuppressive microenvironment [77]. The regulation of estrogen on macrophages needs to be further explored.

Androgens promote macrophage polarization to M2 type by regulating the expression of local cytokines, such as CXCL1 and G-CSF [78]. M2 macrophages form a pro-tumor microenvironment by secreting anti-inflammatory factors (e.g., IL-10) and inhibiting T cell activity [79]. Macrophages are rich in cholesterol and capable of transferring cholesterol into tumor cells [79, 80]. Macrophages promote androgen synthesis by enhancing cholesterol uptake by tumor cells, thereby activating AR signaling in tumor cells [80]. In addition, AR is also expressed in macrophages, and by activating the AR signaling pathway, macrophage-like THP-1 cell lines can upregulate the expression of Triggering Receptor Expressed on Myeloid cells-1 and its downstream cytokines, thereby promoting the migration and invasion of certain tumors such as PCa cells [81]. Androgens not only directly inhibit the bactericidal function of neutrophils (e.g., reduced degranulation and phagocytic activity), but may also indirectly affect the function of other immune cells (e.g., T cells, NK cells) through macrophages [82]. In BCa, androgen-AR signaling not only promotes tumor invasion, but may also recruit immune cells into the tumor microenvironment to form a vicious cycle [22]. This may be one of the important reasons for the significant sex difference in the incidence of BCa.

#### T cells

Estrogen bidirectionally modulates T cell immunity: low levels promote T Helper 1 Cell (Th1) responses, while high levels favor T Helper 2 Cell (Th2) polarization [3, 73]. The regulation of CD8 + T cells by estrogen is bidirectional. On the one hand, estrogen activates the TCR signaling of CD8 + T cells through ERβ and enhances their anti-tumor function. On the other hand, estrogen inhibits the activity of CD8 + T cells by promoting the expansion and function of regulatory T cells (Tregs), thereby weakening the anti-tumor immune response [3, 73]. In urinary tract tumors, lower androgen levels in women and the protective effects of estrogen may work together to reduce tumor aggressiveness [66, 83].

Androgens act directly within CD8 + T cells via AR signaling to regulate Tcf7, a master regulator of T cell differentiation. This drives CD8 + T cells toward an exhausted phenotype (characterized by high PD-1 expression) and impairs their anti-tumor function [5], which is manifested by reduced cytotoxicity and reduced stem-cell-like properties [84]. This inhibitory effect is more pronounced in men, leading directly to accelerated progression of urologic tumors such as BCa and poor response to immunotherapy [85]. Androgen deprivation (e.g., surgical castration or treatment with the small molecule inhibitor abiraterone) has been shown to restore the anti-tumor activity of CD8 + T cells, thereby enhancing the efficacy of PD-1 inhibitors [22, 69]. In addition, the higher ratio of M2 macrophages to Treg in the human male tumor microenvironment indirectly inhibits CD8 + T cell function [74, 86]. This may be one of the important factors in the sex difference in tumor incidence.

##### Limitation and Translational Consideration

Several mechanisms discussed in this review are informed by studies in non-urologic cancers. While these models provide valuable hypotheses, direct translation to bladder or renal cancers requires validation. We have prioritized evidence from urologic malignancies where available and indicate when mechanisms are proposed based on conserved pathways. Furthermore, while we have focused on the most established roles of estrogens and androgens, the potential influence of other hormonal systems (e.g., progesterone, glucocorticoids) on the tumor–stromal interface represents an important area for future research to fully delineate the endocrine landscape of sex disparities.

## Genetic and epigenetic contributions

### X chromosome inactivation and escape genes in cancer susceptibility

In female mammals, X chromosome inactivation (XCI) randomly silences one of the two X chromosomes to achieve dose compensation [87]. Notably, partially evading X-inactivated tumor suppressors (EXITS) such as ATRX, CNKSR2, DDX3X, KDM5C, KDM6, and MAGEC3 remain active on both X chromosomes, thereby reducing cancer risk in women to some extent [88, 89]. This phenomenon suggests that the XCI escape gene plays a non-negligible role in women’s cancer risk. Because there is only one X chromosome in males, a mutation can cause a complete loss of gene function [90]. However, women need two alleles to be inactivated at the same time to be inactivated, which relatively reduces the risk of tumorigenesis. Xiaoxi Li et al. observed a significant increase in tumor incidence due to reduced UTX copy number in a mouse lymphoma model, with a higher incidence of lymphoma in female mice with a single copy of UTX than in double copies, and the highest incidence of tumors in male mice with complete UTX knockout [91]. This negative correlation of gene dose with tumorigenesis can be used as direct evidence to support that UTX is one of the EXITS genes. In women, XCI maintains the biallelic expression of tumor suppressor genes such as KDM6A, forming a double protective effect on cancer. This sex difference not only explains the higher cancer susceptibility in men but also provides a molecular basis for the development of sex-specific precision treatment strategies.

### Y chromosome loss and its oncogenic impact

Y chromosome deletion (LOY) is the most common somatic mutation in male individuals [92]. The biological mechanism of Y chromosome deletion and tumorigenesis deletion or dysfunction of Y chromosome genes, can affect tumorigenesis through a variety of pathways. The incidence of mLOY (mosaic Y chromosome deletion) increases significantly with age (approximately 1.6-fold every 10 years) [93]. Large-scale cohort studies have shown that mLOY carriers have an increased risk of developing solid tumors such as lung cancer and BCa, which may be associated with a chronic inflammatory state due to Y gene deletion [94, 95]. When the Y chromosome is missing, X chromosome homologous genes may emerge, compensated for high expression, but this compensation may be exploited by tumor cells. For example, prostate cancer cells rely on the homologous gene EIF1AX (Eukaryotic Translation Initiation Factor 1 A, X) on the X chromosome to maintain proliferation after the deletion of the Y chromosome, forming a vulnerable target for treatment [96].

Certain genes on the Y chromosome play an important role in regulating the immune response. For example, the UTY (Ubiquitously Transcribed Tetratricopeptide Repeat Gene on Y Chromosome) gene can affect the anti-tumor immune response by regulating macrophage function, and its deletion may lead to weakened macrophage immune surveillance and increased tumor susceptibility [97]. Y chromosome deletion (LOY) in BCa is directly related to T cell dysfunction, leading to an immunosuppressive state in the tumor microenvironment that enhances the aggressiveness of BCa [98]. Y chromosome genomic protein demethylase KDM5D is a transcriptional upregulated gene driven by KRAS-mediated activation of the STAT4 transcription factor, and its deletion in the mouse CRC (colon cancer) model can cause abnormal chromatin modification, leading to down-regulation of the expression of tight junction-related genes between epithelial cells, and promoting the invasion and metastasis of tumor cells [99].

### Non-coding RNAs as sex-specific regulators

XIST (X-inactive specific transcript) acts as a key regulator of XCI through epigenetic modification mechanisms [100]. In hepatocellular carcinoma, the incidence is significantly higher in males than in females, and studies have found that the high expression of the XIST regulator lnc-FTX in females may inhibit tumor progression by stabilizing XIST, while the lack of XIST in males leads to dysregulation of related tumor suppressor genes [100]. This suggests that aberrant expression of XIST may affect sex differences in tumorigenesis. Dysregulation of XIST may also affect tumor prognosis and staging by regulating the expression of tumor-associated genes on the X chromosome [101].

LncRNAs may mediate the regulation of tumor genes by sex hormones, and lncRNAs affect tumor progression by regulating the AR signaling pathway in prostate cancer [102]. circRNAs regulate mRNA stability or translational efficiency, and in breast cancer, circRNAs can affect the splicing and expression of tumor-associated genes by binding to RNA-binding proteins (RBPs) [103]. Some circRNAs may also generate sex-specific cyclic isoforms through reverse splicing, affecting the expression pattern of tumor genes [104]. lncRNAs and circRNAs exhibited sex-specific expression patterns in tumors with significant sex differences [105]. The sex-specific expression patterns, stability, and functional relevance of these RNAs make them promising diagnostic and prognostic biomarker [106–108].

## Microbiome and environmental influences

### Sex-specific urinary microbiota and bladder cancer

Under healthy conditions, significant differences exist in the urinary microbiota composition between males and females. A well-established association has been demonstrated between the female urinary tract microbiota and lower urinary tract symptoms (LUTS), whereas no comparable causal relationship has been confirmed in the male population [109, 110]. Gender disparities in bladder cancer incidence may be attributed not only to variations in hormone levels, genetic predisposition, and behavioral factors, but also to sex-specific distributions of the genitourinary microbiota, potentially constituting an underlying mechanism. Specifically, enrichment of *Actinobacteria* in female urine samples is significantly associated with lower tumor incidence [111], while males exhibit greater microbial diversity and a higher relative abundance of potential pathogens [112]. In patients with NMIBC, a significant correlation is observed between well-differentiated tumor tissue and microbiota diversity, a phenomenon particularly pronounced within the female patient cohort. Notably, these gender-dependent microbial differences are most prominent in the microenvironment of well-differentiated tumors. Female cancer tissues demonstrate a significant increase in the abundance of pro-inflammatory bacterial genera such as Salmonella and Enterobacter, whereas male tissues are characterized by enrichment of opportunistic pathogens including Pseudomona and Acinetobacter [112]. Collectively, these findings suggest that the urinary microbiota may influence bladder cancer tumorigenesis and disease progression through sex-specific immunomodulatory mechanisms and inflammatory cascades [113]. It is important to note that most current evidence is correlative, and a direct causal link between specific microbial profiles and sex-biased cancer incidence remains to be conclusively established.

### Gut microbiota modulates sex-biased anti-tumor immunity

In female mouse models, the gut microbiota is characterized by a predominance of Lachnospiraceae. Under estrogen influence, this microbial profile maintains Th17 cell levels, thereby enhancing the anti-tumor efficacy of immune checkpoint inhibitors (ICIs). Conversely, in male mouse models, the gut microbiota is dominated by Muribaculaceae. Testosterone suppresses Th17 cell differentiation and impairs their immunosuppressive function, which conversely cooperatively enhances the anti-tumor activity of CD8⁺ T cells [6, 114]. Despite exhibiting distinct gut microbiota compositions, male and female mouse models respectively demonstrate anti-tumor immunity through the specific regulation of Th17 and CD8⁺ T cell functions.

## Lifestyle and environmental factors cannot be ignored

### Smoking and chemical exposure

In human society, the prevalence of smoking in men is significantly higher than that in women, and this difference has an important impact on the risk of sex bias in BCa, and smoking is known to be the most well-established risk factor for BCa, with about 50% of BCa cases attributable to smoking [115]. The daily cigarette volume and cumulative smoking index of male smokers were higher than those of women, resulting in the long-term accumulation of carcinogens (such as aromatic amines and polycyclic aromatic hydrocarbons) in tobacco, which can directly stimulate and damage bladder epithelial cells [116]. Male smokers have been shown to have higher levels of N-nitroso compounds in their urine, which directly induce DNA damage in bladder epithelial cells [117]. It is important to note that men have a higher susceptibility to smoking-related genes, resulting in a weaker ability to detoxify aromatic amines in tobacco, which may further increase the risk of BCa [116].

Occupational exposure is the second leading risk factor for BCa, and men are more likely to be exposed to carcinogens due to traditional occupations such as industry and manufacturing, but women may also be at increased risk in certain industries such as textiles [118, 119]. Men are at higher risk of exposure to aromatic amines such as β-naphthylamine and benzidine in industries such as dyes, rubber, and petrochemicals, which can be metabolized by N-hydroxylation to form DNA-reactive intermediates that directly lead to bladder epithelial cell mutations [118].

### Drinking and eating habits

The association between alcohol intake and the risk of urinary tract cancers showed significant sex heterogeneity. Cohort studies have shown an inverted U-shaped association between alcohol consumption and BCa risk in men: light to moderate alcohol consumption (151 to 300 g/week of pure ethanol) significantly increases the risk of developing the disease by 67%, but the risk of excessive alcohol consumption (> 450 g/week) decreases to below baseline [120]. Notably, there was no significant association between alcohol intake and BCa risk in women [121].

In addition, there is a sex bias in the effect of alcohol type on different tumors. Long-term alcohol consumption (40% ≥ethanol) was positively associated with melanoma and basal cell carcinoma risk in men, while only a slightly increased risk of basal cell carcinoma was observed in women [122]. Mechanistic studies have shown that acetaldehyde dehydrogenase (ALDH2) gene polymorphisms (such as rs671) are more likely to lead to acetaldehyde accumulation in males, which promotes mutations through DNA adduct formation. In women, the upregulation of ALDH2 activity by estrogen may partially counteract the genotoxicity of alcohol metabolites [123]. These findings suggest that sex differences in alcohol-related carcinogenic risk are not only due to behavioral factors, but also closely related to the interaction of metabolic pathways and genetic predisposition.

The protective or promoting effect of dietary components on urinary tract tumors is also sex-specific. In a cohort study, total vegetable and non-starchy vegetable intake were significantly inversely associated with BCa risk in women, but no similar association was found in men [124]. A high intake of red/processed meat in men significantly increases the risk of BCa, while a weaker association is associated in women, possibly with androgen-promoting inflammatory effects [125]. In addition, an increase of 100 g of fruit per day in women reduced the risk of non-muscle-invasive bladder cancer (NMIBC) by 28%, while there was no significant protective association in men [126]. These findings suggest that sex differences not only exist in carcinogen exposure levels, but are also deeply rooted in the interaction between nutrient metabolism and molecular regulatory networks, which provides a key basis for the development of sex-stratified dietary intervention strategies.

## Clinical translation and therapeutic strategies

### Sex differences in response to immunotherapy

ICIs exhibit differential effects for treatment in different sexs [127, 128], and this difference may be associated with a stronger T-cell immune response in men, while the immune system in women is more inclined to a regulatory immune response [129]. PD-1 is predominantly expressed on the surface of activated T cells, while PD-L1 is predominantly expressed on tumor cells and antigen-presenting cells [130]. Activation of the PD-1/PD-L1 pathway can inhibit the activity of T cells, thereby helping tumor cells evade the immune system [131]. PD-1 inhibitors have been reported to be more effective in male patients with non-small cell lung cancer (NSCLC), especially in populations with high PD-L1 expression, while other immunological combination strategies may need to be explored in female patients [132].

Several pivotal clinical trials have revealed significant sex-based differences in the efficacy of ICIs among patients with advanced urothelial carcinoma [133]. In the phase III KEYNOTE-045 trial, pembrolizumab demonstrated a clear survival advantage in male patients (HR = 0.73, 95% CI 0.59–0.90), whereas no significant survival benefit was observed in female patients (HR = 0.92, 95% CI 0.63–1.33) [134]. A notable sex imbalance was also present in the first-line cohort of the IMvigor210 trial, where male patients constituted 81% of the enrolled population [135]. This enrollment distribution not only reflects the epidemiological characteristics of bladder cancer but also provides a relevant context for assessing potential sex-associated disparities in treatment outcomes [135]. These findings are consistent with a large meta-analysis that confirmed significantly better survival in male patients treated with PD-1/PD-L1 inhibitors compared to females (*P* = 0.0019), with pooled hazard ratios of 0.72 (95% CI 0.65–0.79) for men and 0.86 (95% CI 0.79–0.93) for women [133]. The concordance across studies underscores sex as a crucial biological variable influencing response to immunotherapy in bladder cancer. The underlying mechanism may be attributed to the significant role of the androgen–androgen receptor signaling axis in shaping a sex-dimorphic tumor immune microenvironment. This pathway promotes the differentiation of CD8⁺ T cells toward an exhausted phenotype in males and upregulates the expression of immune checkpoint molecules such as PD-1, collectively fostering a more immunosuppressive microenvironment [5]. In summary, accumulating evidence strongly indicates that patient sex is a key biological variable affecting responses to immunotherapy. Future clinical practice and trial designs should incorporate sex as an important factor in patient stratification and therapeutic decision-making.

### Targeting hormonal pathways in urologic cancers

Sex hormone signaling pathways are known to play a key role in sex differences in a variety of cancers, and the sex of patients needs to be fully considered in these treatments that target the signaling pathways. Male patients with BCa benefit more from AR signaling inhibition, while female patients are more concerned about estrogen receptor-related pathways [136], Targeting ARs demonstrates promising therapeutic potential [137]. Preclinical and early-phase clinical studies indicate that AR antagonists may inhibit tumor progression directly while also reshaping the immune microenvironment. Their combination with immune checkpoint inhibitors could enhance T-cell activity via ADT, thereby producing synergistic antitumor effects [137, 138]. In female patients and specific subtypes of prostate cancer unique to males, modulation of ER signaling has emerged as another critical focus [139, 140]. However, the biological role of ERβ in bladder cancer remains controversial; the complexity of its mechanistic actions significantly influences the strategic direction of targeted therapy development. Existing data suggest that ERβ may facilitate proliferation and invasion of bladder cancer cells by regulating the miR-92a/DAB2IP signaling axis, implying a potential oncogenic role [141]. Nevertheless, the proposed “tumor suppressor” or immunomodulatory properties observed in other malignancies remain insufficiently substantiated in bladder cancer models [142].

Amidst these uncertainties, current therapeutic strategies targeting ERβ exhibit marked asymmetry. Present research primarily emphasizes ERβ inhibition, utilizing SERMs to overcome cisplatin resistance in bladder cancer [143]. Conversely, therapeutic approaches aimed at harnessing ERβ’s putative tumor-suppressive or immunomodulatory functions through agonism remain largely exploratory, hindered by a lack of robust supporting evidence, and their clinical viability remains undefined. A central unresolved question is whether ERβ acts as a promoter or suppressor of tumorigenesis in bladder cancer. Future investigations must aim to elucidate its precise functional role, thereby laying a theoretical foundation and guiding translational efforts toward precision-targeted therapies.

### Gender-specific therapeutic targets LOY and KDM6A

The loss of the Y chromosome (LOY) is not only a common occurrence in male cancers but also a critical driver of immunosuppressive tumor microenvironment formation. This underscores the potential of targeting LOY-related pathways as a novel, male-centric therapeutic approach [144]. Such strategies may include restoring the function of Y-linked tumor suppressor genes or blocking LOY-induced immunosuppression [145]. A compelling application of this principle is the combination of immunotherapy with gender-based patient stratification; specifically, prioritizing PD-1 or PD-L1 inhibitors in male patients with documented LOY holds promise for improving treatment outcomes by mitigating the distinct immunological and genomic vulnerabilities characteristic of male cancers [144].

Compounding this, gender disparities in cancer susceptibility are further exemplified by KDM6A, an X-linked gene whose expression profile is markedly influenced by sex. While females carry two KDM6A alleles, X-chromosome inactivation limits its expression to approximately half of their cells. In contrast, males, with only a single functional KDM6A copy, face a heightened risk of functional compromise [146]. This genetic asymmetry likely underpins the elevated incidence of specific cancers, including bladder and colorectal cancer, in men [146, 147]. At a mechanistic level, KDM6A loss perturbs the balance between activating COMPASS and repressive polycomb complexes, thereby remodeling chromatin and promoting the expression of oncogenic drivers such as IGF1 and ELN [147]. Together, these findings lay a solid theoretical groundwork for advancing male-specific cancer therapies.

### Gaps in the development of sex-specific biomarkers

Current research has yet to establish a systematic and in-depth understanding of the biological mechanisms underlying sex-based differences in disease, a knowledge gap that is particularly evident in non-reproductive cancers, neurodegenerative disorders, and autoimmune conditions [148]. Compounding this issue, the frequent oversight of sex as a biological variable in study design, coupled with insufficient application of sophisticated analytical methods, has resulted in a persistent shortage of rigorously validated and clinically actionable sex-specific biomarkers [149]. This critical deficit ultimately impedes the development and implementation of sex-informed strategies for precision prevention, diagnosis, and treatment.

## Conclusions and prospects

Urinary cancers demonstrate significant sex-based disparities, with males generally exhibiting higher morbidity and mortality rates. These differences arise from a complex interplay between intrinsic biological mechanisms and extrinsic environmental factors. Key contributing mechanisms include the divergent roles of sex hormones—where androgens frequently promote tumor progression, whereas estrogens may confer protective effects—as well as genetic and epigenetic influences, such as biallelic expression of X‑linked tumor suppressors in females and loss of the Y chromosome in males. Furthermore, sex‑specific modulation of the immune microenvironment and microbiota profoundly influences tumor development and therapeutic response. Clinically, these biological distinctions directly shape treatment outcomes, as evidenced by sex‑stratified efficacy data from pivotal immunotherapy trials. Therefore, incorporating sex as a fundamental biological variable into research frameworks, therapeutic strategies, and public health initiatives is essential for advancing precision oncology.

To further the development of sex‑informed precision oncology, coordinated efforts across multiple domains will be required. Future research should prioritize elucidating the complex interactions between sex hormones and sex chromosomes, and rigorously validate emerging sex‑specific therapeutic targets, such as the androgen receptor pathway and Y‑chromosome loss mechanisms in males. Translational efforts must focus on developing sex‑stratified immunotherapeutic approaches and clinically applicable biomarkers, while ensuring sex‑balanced enrollment and pre‑specified sex‑based analyses in clinical trials. At a systemic level, establishing dedicated sex‑specific biobanks and integrating multi‑omics datasets will provide crucial infrastructure. Ultimately, only by embedding the biology of sex differences throughout the continuum of cancer research and clinical care can we achieve truly personalized, equitable, and effective oncology practice.

## Supplementary Information

Supplementary Material 1

## Data Availability

The data used to generate Figure 1 are derived from the following source: [2].

## Abbreviations

BCa: Bladder cancer
AR: Androgen receptor
DHT: Dihydrotestosterone
EPPK1: Epiplakin1
JUP: Junction plakoglobin
MAPK: Mitogen-activated protein kinase
TGF-β: Transforming growth factor beta
TβRI: TGF-β receptor I
IGF1: Insulin-like growth factor 1
PARP: Poly ADP-ribose polymerase
BER: Base excision repair
NHEJ: Non-homologous end joining
ERα: Estrogen receptor alpha
ERβ: Estrogen receptor beta
SERM: Selective estrogen receptor modulator
MIBC: Muscle-invasive bladder cancer
NMIBC: Non-muscle-invasive bladder cancer
PD-1: Programmed cell death protein 1
PD-L1: Programmed death-ligand 1
ADT: Androgen deprivation therapy
GPER1: G protein-coupled estrogen receptor 1
VEGF: Vascular endothelial growth factor
Th1: T helper 1 cell
Th2: T helper 2 cell
Treg: Regulatory T cell
XCI: X chromosome inactivation
EXITS: Escape from X-inactivation tumor suppressors
LOY: Loss of Y chromosome
mLOY: Mosaic loss of Y chromosome
CRC: Colon cancer
XIST: X-inactive specific transcript
HCC: Hepatocellular carcinoma
lncRNA: Long non-coding RNA
circRNA: Circular RNA
LUTS: Lower urinary tract symptoms
ICI: Immune checkpoint inhibitor
NSCLC: Non-small cell lung cancer
ALDH2: Aldehyde dehydrogenase 2

## References

[1] Schafer EJ, Jemal A, Wiese D, et al. Disparities and trends in genitourinary cancer incidence and mortality in the USA. Eur Urol. 2023;84(1):117–26. 10.1016/j.eururo.2022.11.023. 36566154 10.1016/j.eururo.2022.11.023

[2] Siegel RL, Kratzer TB, Giaquinto AN, Sung H, Jemal A. Cancer statistics, 2025. Ca. 2025;75(1):10. 10.3322/caac.21871. 39817679 10.3322/caac.21871 PMC11745215

[3] Conforti F, Pala L, Di Mitri D, et al. Sex hormones, the anticancer immune response, and therapeutic opportunities. Cancer Cell. 2025;43(3):343–60. 10.1016/j.ccell.2025.02.013. 40068594 10.1016/j.ccell.2025.02.013

[4] Wang L, Shilatifard A. UTX mutations in human cancer. Cancer Cell. 2019;35(2):168–76. 10.1016/j.ccell.2019.01.001. 30753822 10.1016/j.ccell.2019.01.001 PMC6589339

[5] Kwon H, Schafer JM, Song NJ, et al. Androgen conspires with the CD8 + T cell exhaustion program and contributes to sex bias in cancer. Sci Immunol. 2022;7(73):eabq2630. 10.1126/sciimmunol.abq2630. 35420889 10.1126/sciimmunol.abq2630 PMC9374385

[6] Wang J, Li D, Wu R, Feng D. Cutting-edge advancements in the antibiotics‐gut microbiota‐urinary tumour axis. Cell Prolif. 2025;58(5):e70023. 10.1111/cpr.70023. 40091493 10.1111/cpr.70023 PMC12099210

[7] Prall OW, Sarcevic B, Musgrove EA, Watts CK, Sutherland RL. Estrogen-induced activation of Cdk4 and Cdk2 during G1-S phase progression is accompanied by increased cyclin D1 expression and decreased cyclin-dependent kinase inhibitor association with cyclin E-Cdk2. J Biol Chem. 1997;272(16):10882–94. 10.1074/jbc.272.16.10882. 9099745 10.1074/jbc.272.16.10882

[8] Rim EY, Clevers H, Nusse R. The wnt pathway: from signaling mechanisms to synthetic modulators. Annu Rev Biochem. 2022;91:571–98. 10.1146/annurev-biochem-040320-103615. 35303793 10.1146/annurev-biochem-040320-103615

[9] Yedidia-Aryeh L, Goldberg M. The interplay between the cellular response to DNA double-strand breaks and estrogen. Cells. 2022;11(19): 3097. 10.3390/cells11193097. 36231059 10.3390/cells11193097 PMC9563627

[10] Abdel-Hafiz HA, Schafer JM, Chen X, et al. Y chromosome loss in cancer drives growth by evasion of adaptive immunity. Nature. 2023;619(7970):624–31. 10.1038/s41586-023-06234-x. 37344596 10.1038/s41586-023-06234-x PMC10975863

[11] Ece G, Aktaş A, Caner A, et al. The urogenital system microbiota: is it a new gamechanger in urogenital cancers? Microorganisms. 2025;13(2): 315. 10.3390/microorganisms13020315. 40005682 10.3390/microorganisms13020315 PMC11858393

[12] Kouzmenko AP, Takeyama K, Ito S, et al. Wnt/β-Catenin and Estrogen Signaling Converge in Vivo*. J Biol Chem. 2004;279(39):40255–8. 10.1074/jbc.C400331200. 15304487 10.1074/jbc.C400331200

[13] Somjen D, Weisman Y, Kohen F, et al. 25-hydroxyvitamin D3-1alpha-hydroxylase is expressed in human vascular smooth muscle cells and is upregulated by parathyroid hormone and estrogenic compounds. Circulation. 2005;111(13):1666–71. 10.1161/01.CIR.0000160353.27927.70. 15795327 10.1161/01.CIR.0000160353.27927.70

[14] Corachán A, Ferrero H, Aguilar A, et al. Inhibition of tumor cell proliferation in human uterine leiomyomas by vitamin D via Wnt/β-catenin pathway. Fertil Steril. 2019;111(2):397–407. 10.1016/j.fertnstert.2018.10.008. 30458994 10.1016/j.fertnstert.2018.10.008

[15] Krishnan AV, Swami S, Feldman D. Vitamin D and breast cancer: Inhibition of estrogen synthesis and signaling. J Steroid Biochem Mol Biol. 2010;121(1):343–8. 10.1016/j.jsbmb.2010.02.009. 20156557 10.1016/j.jsbmb.2010.02.009

[16] Chen CR, Kang Y, Siegel PM, Massagué J. E2F4/5 and p107 as Smad Cofactors Linking the TGFβ Receptor to c-myc Repression. Cell. 2002;110(1):19–32. 10.1016/S0092-8674(02)00801-2. 12150994 10.1016/s0092-8674(02)00801-2

[17] Wang Y, Tong X, Xiao Y, et al. Regulating integrin β1 to restore gonadotropin-releasing hormone–tanycyte unit function in polycystic ovary syndrome-related hypothalamic dysregulation. Research. 2025;8:0619. 10.34133/research.0619. 39975575 10.34133/research.0619 PMC11836200

[18] Seoane J, Gomis RR. TGF-β family signaling in tumor suppression and cancer progression. Cold Spring Harb Perspect Biol. 2017;9(12):a022277. 10.1101/cshperspect.a022277. 28246180 10.1101/cshperspect.a022277 PMC5710110

[19] Alsamraae M, Costanzo-Garvey D, Teply BA, et al. Androgen receptor inhibition suppresses anti-tumor neutrophil response against bone metastatic prostate cancer via regulation of TβRI expression. Cancer Lett. 2023;579:216468. 10.1016/j.canlet.2023.216468. 37940068 10.1016/j.canlet.2023.216468 PMC10710875

[20] Yang L, Huang W, Bai X, et al. Androgen dihydrotestosterone promotes bladder cancer cell proliferation and invasion via EPPK1-mediated MAPK/JUP signalling. Cell Death Dis. 2023;14(6):363. 10.1038/s41419-023-05882-1. 37328487 10.1038/s41419-023-05882-1 PMC10275919

[21] Hu Y, Wu K, Liu Y, et al. LY6/PLAUR domain containing 3 (LYPD3) maintains melanoma cell stemness and mediates an immunosuppressive microenvironment. Biol Direct. 2023;18:72. 10.1186/s13062-023-00424-3. 37924160 10.1186/s13062-023-00424-3 PMC10623712

[22] Chen J, Huang CP, Quan C, et al. The androgen receptor in bladder cancer. Nat Rev Urol. 2023;20(9):560–74. 10.1038/s41585-023-00761-y. 37072491 10.1038/s41585-023-00761-y

[23] Wu J, Li J, Liu Y, et al. Tannic acid repair of zearalenone-induced damage by regulating the death receptor and mitochondrial apoptosis signaling pathway in mice. Environ Pollut. 2021;287:117557. 10.1016/j.envpol.2021.117557. 34167001 10.1016/j.envpol.2021.117557

[24] Adzavon YM, Culig Z, Sun Z. Interactions between androgen and IGF1 axes in prostate tumorigenesis. Nat Rev Urol. 2024. 10.1038/s41585-024-00942-3. 39375467 10.1038/s41585-024-00942-3

[25] Westaby D, Fenor de La Maza M, Paschalis A, et al. A new old target: androgen receptor signaling and advanced prostate cancer. Annu Rev Pharmacol Toxicol. 2022;62:131–53. 10.1146/annurev-pharmtox-052220-015912. 34449248 10.1146/annurev-pharmtox-052220-015912

[26] Gilbert S, Péant B, Malaquin N, et al. Targeting IKKε in androgen-independent prostate cancer causes phenotypic senescence and genomic instability. Mol Cancer Ther. 2022;21(3):407–18. 10.1158/1535-7163.MCT-21-0519. 34965959 10.1158/1535-7163.MCT-21-0519 PMC9377745

[27] Zhao S, Li P, Wu W, et al. Roles of ferroptosis in urologic malignancies. Cancer Cell Int. 2021;21:676. 10.1186/s12935-021-02264-5. 34922551 10.1186/s12935-021-02264-5 PMC8684233

[28] Cheng W, Chen W, Jia R. The role of pyroptosis in the progression and targeted therapeutic approaches for urological malignancies. J Inflamm Res. 2024;17:9567–81. 10.2147/JIR.S487740. 39606639 10.2147/JIR.S487740 PMC11600921

[29] Liu Y, Lu S, Wu L, Yang L, Yang L, Wang J. The diversified role of mitochondria in ferroptosis in cancer. Cell Death Dis. 2023;14(8):519. 10.1038/s41419-023-06045-y. 37580393 10.1038/s41419-023-06045-y PMC10425449

[30] Lai W, Chen J, Wang T, Liu Q. Crosstalk between ferroptosis and steroid hormone signaling in gynecologic cancers. Front Mol Biosci. 2023;10:1223493. 10.3389/fmolb.2023.1223493. 37469703 10.3389/fmolb.2023.1223493 PMC10352791

[31] Lv D, Shi Y, Kou L, Zhang D, Guo Y, Zhao S. Precision targeting of androgen receptor-ferroptosis crosstalk in prostate cancer: from mechanisms to therapeutic strategies. Pharmacol Res. 2025;219:107915. 10.1016/j.phrs.2025.107915. 40819692 10.1016/j.phrs.2025.107915

[32] Ma W, Jiang X, Jia R, Li Y. Mechanisms of ferroptosis and targeted therapeutic approaches in urological malignancies. Cell Death Discov. 2024;10:432. 10.1038/s41420-024-02195-w. 39384767 10.1038/s41420-024-02195-w PMC11464522

[33] Chen H, Lyu F, Gao X. Advances in ferroptosis for castration-resistant prostate cancer treatment: novel drug targets and combination therapy strategies. Prostate Cancer Prostatic Dis. 2024. 10.1038/s41391-024-00933-w. 39733054 10.1038/s41391-024-00933-w PMC12909126

[34] Shi J, Gao W, Shao F. Pyroptosis: gasdermin-mediated programmed necrotic cell death. Trends Biochem Sci. 2017;42(4):245–54. 10.1016/j.tibs.2016.10.004. 27932073 10.1016/j.tibs.2016.10.004

[35] Loveless R, Bloomquist R, Teng Y. Pyroptosis at the forefront of anticancer immunity. J Exp Clin Cancer Res. 2021;40:264. 10.1186/s13046-021-02065-8. 34429144 10.1186/s13046-021-02065-8 PMC8383365

[36] Loveless R, Bloomquist R, Teng Y. Pyroptosis at the forefront of anticancer immunity. J Exp Clin Cancer Res CR. 2021;40: 264. 10.1186/s13046-021-02065-8. 34429144 10.1186/s13046-021-02065-8 PMC8383365

[37] Vona R, Cittadini C, Ortona E, Matarrese P. Sex disparity in cancer: role of autophagy and estrogen receptors. Cells. 2025;14(4):273. 10.3390/cells14040273. 39996745 10.3390/cells14040273 PMC11854201

[38] Zhao Y, Klionsky DJ, Wang X, Huang Q, Deng Z, Xiang J. The estrogen–autophagy axis: insights into cytoprotection and therapeutic potential in cancer and infection. Int J Mol Sci. 2024;25(23):12576. 10.3390/ijms252312576. 39684286 10.3390/ijms252312576 PMC11641569

[39] Komarla A, Dufresne S, Towers CG. Recent advances in the role of autophagy in endocrine-dependent tumors. Endocr Rev. 2023;44(4):629–46. 10.1210/endrev/bnad001. 36631217 10.1210/endrev/bnad001 PMC10335171

[40] Wu Q, Sharma D. Autophagy and breast cancer: connected in growth, progression, and therapy. Cells. 2023;12(8):1156. 10.3390/cells12081156. 37190065 10.3390/cells12081156 PMC10136604

[41] Siatis KE, Giannopoulou E, Manou D, et al. Resistance to hormone therapy in breast cancer cells promotes autophagy and EGFR signaling pathway. Am J Physiol Cell Physiol. 2023;325(3):C708. 10.1152/ajpcell.00199.2023. 37575061 10.1152/ajpcell.00199.2023 PMC10625825

[42] Rajan A, Varghese GR, Yadev I, et al. Modulation of BRCA1 mediated DNA damage repair by deregulated ER-α signaling in breast cancers. Am J Cancer Res. 2022;12(1):17–47. 35141003 PMC8822286

[43] Rangsrikitphoti P, Marquez-Garban DC, Pietras RJ, McGowan E, Boonyaratanakornkit V. Sex steroid hormones and DNA repair regulation: implications on cancer treatment responses. J Steroid Biochem Mol Biol. 2023;227:106230. 10.1016/j.jsbmb.2022.106230. 36450315 10.1016/j.jsbmb.2022.106230

[44] Wang J, Li C, Han J, et al. Reassessing the roles of oxidative DNA base lesion 8-oxoGua and repair enzyme OGG1 in tumorigenesis. J Biomed Sci. 2025;32:1. 10.1186/s12929-024-01093-8. 39741341 10.1186/s12929-024-01093-8 PMC11689541

[45] Bhardwaj P, Iyengar NM, Zahid H, et al. Obesity promotes breast epithelium DNA damage in women carrying a germline mutation in BRCA1 or BRCA2. Sci Transl Med. 2023;15(684):eade1857. 10.1126/scitranslmed.ade1857. 36812344 10.1126/scitranslmed.ade1857 PMC10557057

[46] Wu W, Zhu J, Nihira NT, et al. Ribosomal S6 kinase (RSK) plays a critical role in DNA damage response via the phosphorylation of histone lysine demethylase KDM4B. Breast Cancer Res. 2024;26:146. 10.1186/s13058-024-01901-x. 39434131 10.1186/s13058-024-01901-x PMC11492477

[47] Ueda T, Kanai A, Komuro A, et al. KDM4B promotes acute myeloid leukemia associated with AML1-ETO by regulating chromatin accessibility. FASEB Bioadv. 2021;3(12):1020–33. 10.1096/fba.2021-00030. 34938963 10.1096/fba.2021-00030 PMC8664044

[48] Shafi AA, McNair CM, McCann JJ, et al. The circadian cryptochrome, CRY1, is a pro-tumorigenic factor that rhythmically modulates DNA repair. Nat Commun. 2021;12:401. 10.1038/s41467-020-20513-5. 33452241 10.1038/s41467-020-20513-5 PMC7810852

[49] Hasterok S, Scott TG, Roller DG, et al. The androgen receptor does not directly regulate the transcription of DNA damage response genes. Mol Cancer Res. 2023;21(12):1329–41. 10.1158/1541-7786.MCR-23-0358. 37698543 10.1158/1541-7786.MCR-23-0358 PMC11022999

[50] Ganguly S, Lone Z, Muskara A, et al. Intratumoral androgen biosynthesis associated with 3β-hydroxysteroid dehydrogenase 1 promotes resistance to radiotherapy in prostate cancer. J Clin Invest. 2023;133(22):e165718. 10.1172/JCI165718. 37966114 10.1172/JCI165718 PMC10645386

[51] Talazoparib plus enzalutamide. in men with first-line metastatic castration-resistant prostate cancer (TALAPRO-2): a randomised, placebo-controlled, phase 3 trial. Lancet. 2023;402(10398):291–303. 10.1016/S0140-6736(23)01055-3. 37285865 10.1016/S0140-6736(23)01055-3

[52] Teng J, Wang ZY, Jarrard DF, Bjorling DE. Roles of estrogen receptor α and β in modulating urothelial cell proliferation. Endocr Relat Cancer. 2008;15(1):351–64. 10.1677/erc.1.01255. 18310301 10.1677/erc.1.01255 PMC3513362

[53] Ide H, Inoue S, Miyamoto H. Histopathological and prognostic significance of the expression of sex hormone receptors in bladder cancer: a meta-analysis of immunohistochemical studies. PLoS One. 2017;12(3):e0174746. 10.1371/journal.pone.0174746. 28362839 10.1371/journal.pone.0174746 PMC5375178

[54] Belluti S, Imbriano C, Casarini L. Nuclear estrogen receptors in prostate cancer: from genes to function. Cancers (Basel). 2023;15(18):4653. 10.3390/cancers15184653. 37760622 10.3390/cancers15184653 PMC10526871

[55] Di Zazzo E, Galasso G, Giovannelli P, et al. Estrogen receptors in epithelial-mesenchymal transition of prostate cancer. Cancers (Basel). 2019;11(10):1418. 10.3390/cancers11101418. 31548498 10.3390/cancers11101418 PMC6826537

[56] Li J, Liu Q, Jiang C. Signal crosstalk and the role of estrogen receptor beta (ERβ) in prostate cancer. Med Sci Monit. 2022;28:e935599-1-e935599-7. 10.12659/MSM.935599. 35383138 10.12659/MSM.935599 PMC8996693

[57] Ramírez-de-Arellano A, Pereira-Suárez AL, Rico-Fuentes C, López-Pulido EI, Villegas-Pineda JC, Sierra-Diaz E. Distribution and effects of estrogen receptors in prostate cancer: associated molecular mechanisms. Front Endocrinol (Lausanne). 2022;12:811578. 10.3389/fendo.2021.811578. 35087479 10.3389/fendo.2021.811578 PMC8786725

[58] Yan S, Wang J, Chen H, Zhang D, Imam M. Divergent features of ERβ isoforms in triple negative breast cancer: progress and implications for further research. Front Cell Dev Biol. 2023;11:1240386. 10.3389/fcell.2023.1240386. 37936981 10.3389/fcell.2023.1240386 PMC10626554

[59] Jefferi NE, Shamhari AA, Noor Azhar NK, et al. The role of ERα and ERβ in castration-resistant prostate cancer and current therapeutic approaches. Biomedicines. 2023;11(3):826. 10.3390/biomedicines11030826. 36979805 10.3390/biomedicines11030826 PMC10045750

[60] Young GM, Helms TH, Kulp SK, et al. OSU-ERβ-12: a promising pre-clinical candidate selective estrogen receptor beta agonist. Sci Rep. 2025;15:38377. 10.1038/s41598-025-22258-x. 41184385 10.1038/s41598-025-22258-x PMC12583746

[61] Elahi Najafi MA, Matsukawa T, Miyamoto H. Recent advances in understanding the role of sex hormone receptors in urothelial cancer. Oncol Res. 33(6):1255–70. 10.32604/or.2025.062142 10.32604/or.2025.062142 PMC12144608 40486871

[62] Liu Y, Ma H, Yao J. ER&alpha;, A Key Target for Cancer Therapy: A Review. OTT. 2020;13:2183–91. 10.2147/OTT.S236532. 10.2147/OTT.S236532 PMC7073439 32210584

[63] Sikic D, Taubert H, Wirtz RM, et al. High androgen receptor mRNA expression is associated with improved outcome in patients with high-risk non-muscle-invasive bladder cancer. Life (Basel). 2021;11(7):642. 10.3390/life11070642. 34209360 10.3390/life11070642 PMC8306811

[64] Sottnik JL, Vanderlinden L, Joshi M, et al. Androgen receptor regulates CD44 expression in bladder cancer. Cancer Res. 2021;81(11):2833–46. 10.1158/0008-5472.CAN-20-3095. 33687952 10.1158/0008-5472.CAN-20-3095 PMC8782536

[65] De Ieso ML, Aldoghachi AF, Tilley WD, Dwyer AR. Are androgen receptor agonists a treatment option in bladder cancer? J Steroid Biochem Mol Biol. 2025;245:106623. 10.1016/j.jsbmb.2024.106623. 39306143 10.1016/j.jsbmb.2024.106623

[66] Sun A, Luo Y, Xiao W, et al. Androgen receptor transcriptionally inhibits programmed death ligand-1 expression and influences immune escape in bladder cancer. Lab Invest. 2023. 10.1016/j.labinv.2023.100148. 37059268 10.1016/j.labinv.2023.100148

[67] Lim CL, Or YZ, Ong Z, et al. Estrogen exacerbates mammary involution through neutrophil-dependent and -independent mechanism. eLife. 2020;9:e57274. 10.7554/eLife.57274. 32706336 10.7554/eLife.57274 PMC7417171

[68] Yajuk O, Baron M, Toker S, Zelter T, Fainsod-Levi T, Granot Z. The PD-L1/PD-1 axis blocks neutrophil cytotoxicity in cancer. Cells. 2021;10(6):1510. 10.3390/cells10061510. 34203915 10.3390/cells10061510 PMC8232689

[69] Zhang X, Cheng L, Gao C, et al. Androgen signaling contributes to sex differences in cancer by inhibiting NF-κB activation in T cells and suppressing antitumor immunity. Cancer Res. 2023;83(6):906–21. 10.1158/0008-5472.CAN-22-2405. 36634207 10.1158/0008-5472.CAN-22-2405

[70] Pala L, De Pas T, Conforti F. Boosting anticancer immunotherapy through androgen receptor blockade. Cancer Cell. 2022;40(5):455–7. 10.1016/j.ccell.2022.04.007. 35537411 10.1016/j.ccell.2022.04.007

[71] Chen S, Saeed AFUH, Liu Q, et al. Macrophages in immunoregulation and therapeutics. Signal Transduct Target Ther. 2023;8:207. 10.1038/s41392-023-01452-1. 37211559 10.1038/s41392-023-01452-1 PMC10200802

[72] Locati M, Curtale G, Mantovani A. Diversity, mechanisms and significance of macrophage plasticity. Annu Rev Pathol. 2020;15:123–47. 10.1146/annurev-pathmechdis-012418-012718. 31530089 10.1146/annurev-pathmechdis-012418-012718 PMC7176483

[73] Artham S, Chang CY, McDonnell DP. Eosinophilia in cancer and its regulation by sex hormones. Trends Endocrinol Metab. 2023;34(1):5–20. 10.1016/j.tem.2022.11.002. 36443206 10.1016/j.tem.2022.11.002 PMC10122120

[74] Yang Y, Wang Y, Zou H, et al. GPER1 signaling restricts macrophage proliferation and accumulation in human hepatocellular carcinoma. Front Immunol. 2024;15:1481972. 10.3389/fimmu.2024.1481972. 39582864 10.3389/fimmu.2024.1481972 PMC11582010

[75] Adachi A, Honda T, Egawa G, et al. Estradiol suppresses psoriatic inflammation in mice by regulating neutrophil and macrophage functions. J Allergy Clin Immunol. 2022;150(4):909-919.e8. 10.1016/j.jaci.2022.03.028. 35589416 10.1016/j.jaci.2022.03.028

[76] Barcena ML, Christiansen-Mensch C, Aslam M, Haritonow N, Ladilov Y, Regitz-Zagrosek V. Upregulation of mitochondrial Sirt3 and alleviation of the inflammatory phenotype in macrophages by estrogen. Cells. 2024;13(17):1420. 10.3390/cells13171420. 39272992 10.3390/cells13171420 PMC11393879

[77] Bakhshi P, Ho JQ, Zanganeh S. Sex-specific outcomes in cancer therapy: the central role of hormones. Front Med Technol. 2024;6:1320690. 10.3389/fmedt.2024.1320690. 38362126 10.3389/fmedt.2024.1320690 PMC10867131

[78] Hreha TN, Collins CA, Daugherty AL, Griffith JM, Hruska KA, Hunstad DA. Androgen-influenced polarization of activin A-producing macrophages accompanies post-pyelonephritic renal scarring. Front Immunol. 2020;11:1641. 10.3389/fimmu.2020.01641. 32849562 10.3389/fimmu.2020.01641 PMC7399094

[79] Rehman A, Pacher P, Haskó G. Role of macrophages in the endocrine system. Trends Endocrinol Metab. 2021;32(4):238–56. 10.1016/j.tem.2020.12.001. 33455863 10.1016/j.tem.2020.12.001

[80] El-Kenawi A, Dominguez-Viqueira W, Liu M, et al. Macrophage-derived cholesterol contributes to therapeutic resistance in prostate cancer. Cancer Res. 2021;81(21):5477–90. 10.1158/0008-5472.CAN-20-4028. 34301759 10.1158/0008-5472.CAN-20-4028 PMC8563406

[81] Cioni B, Zaalberg A, van Beijnum JR, et al. Androgen receptor signalling in macrophages promotes TREM-1-mediated prostate cancer cell line migration and invasion. Nat Commun. 2020;11:4498. 10.1038/s41467-020-18313-y. 32908142 10.1038/s41467-020-18313-y PMC7481219

[82] Hreha TN, Collins CA, Cole EB, Jin RJ, Hunstad DA. Androgen exposure impairs neutrophil maturation and function within the infected kidney. mBio. 2024;15(2):e03170-23. 10.1128/mbio.03170-23. 38206009 10.1128/mbio.03170-23 PMC10865792

[83] Deltourbe L, Lacerda Mariano L, Hreha TN, Hunstad DA, Ingersoll MA. The impact of biological sex on diseases of the urinary tract. Mucosal Immunol. 2022;15(5):857–66. 10.1038/s41385-022-00549-0. 35869147 10.1038/s41385-022-00549-0 PMC9305688

[84] Yang C, Jin J, Yang Y, et al. Androgen receptor-mediated CD8 + T cell stemness programs drive sex differences in antitumor immunity. Immunity. 2022;55(7):1268-1283.e9. 10.1016/j.immuni.2022.05.012. 35700739 10.1016/j.immuni.2022.05.012

[85] Androgen Receptor Signaling Reduces Male Antitumor CD8 + T-cell Activity. Cancer Discov. 2022;12(8):1836. 10.1158/2159-8290.CD-RW2022-117. 10.1158/2159-8290.CD-RW2022-117 35748591

[86] Pinto JA, Araujo JM, Gómez HL. Sex, immunity, and cancer. Biochimica et Biophysica Acta (BBA) - Reviews on Cancer. 2022;1877(1):188647. 10.1016/j.bbcan.2021.188647. 34767966 10.1016/j.bbcan.2021.188647

[87] Caramia F, Speed TP, Shen H, Haupt Y, Haupt S. Establishing the link between X-chromosome aberrations and TP53 status, with breast cancer patient outcomes. Cells. 2023;12(18):2245. 10.3390/cells12182245. 37759468 10.3390/cells12182245 PMC10526523

[88] Wang D, Tang L, Wu Y, et al. Abnormal X chromosome inactivation and tumor development. Cell Mol Life Sci. 2020;77(15):2949–58. 10.1007/s00018-020-03469-z. 32040694 10.1007/s00018-020-03469-z PMC11104905

[89] Dunford A, Weinstock DM, Savova V, et al. Tumor suppressor genes that escape from X-inactivation contribute to cancer sex bias. Nat Genet. 2017;49(1):10–6. 10.1038/ng.3726. 27869828 10.1038/ng.3726 PMC5206905

[90] Cáceres A, Jene A, Esko T, Pérez-Jurado LA, González JR. Extreme downregulation of chromosome Y and cancer risk in men. J Natl Cancer Inst. 2020;112(9):913–20. 10.1093/jnci/djz232. 31945786 10.1093/jnci/djz232 PMC7492764

[91] Li X, Zhang Y, Zheng L, Liu M, Chen CD, Jiang H. UTX is an escape from X-inactivation tumor-suppressor in B cell lymphoma. Nat Commun. 2018;9:2720. 10.1038/s41467-018-05084-w. 30006524 10.1038/s41467-018-05084-w PMC6045675

[92] Bruhn-Olszewska B, Markljung E, Rychlicka-Buniowska E, Sarkisyan D, Filipowicz N, Dumanski JP. The effects of loss of Y chromosome on male health. Nat Rev Genet. 2025;26(5):320–35. 10.1038/s41576-024-00805-y. 39743536 10.1038/s41576-024-00805-y

[93] Loftfield E, Zhou W, Yeager M, Chanock SJ, Freedman ND, Machiela MJ. Mosaic Y loss is moderately associated with solid tumor risk. Cancer Res. 2019;79(3):461–6. 10.1158/0008-5472.CAN-18-2566. 30510122 10.1158/0008-5472.CAN-18-2566 PMC6359954

[94] Qin N, Li N, Wang C, et al. Association of Mosaic Loss of Chromosome Y with Lung Cancer Risk and Prognosis in a Chinese Population. J Thorac Oncol. 2019;14(1):37–44. 10.1016/j.jtho.2018.09.013. 30267841 10.1016/j.jtho.2018.09.013

[95] Wright DJ, Day FR, Kerrison ND, et al. Genetic variants associated with mosaic Y chromosome loss highlight cell cycle genes and overlap with cancer susceptibility. Nat Genet. 2017;49(5):674–9. 10.1038/ng.3821. 28346444 10.1038/ng.3821 PMC5973269

[96] Köferle A, Schlattl A, Hörmann A, et al. Interrogation of cancer gene dependencies reveals paralog interactions of autosome and sex chromosome-encoded genes. Cell Rep. 2022. 10.1016/j.celrep.2022.110636. 35417719 10.1016/j.celrep.2022.110636

[97] Maan AA, Eales J, Akbarov A, et al. The Y chromosome: a blueprint for men’s health? Eur J Hum Genet. 2017;25(11):1181–8. 10.1038/ejhg.2017.128. 28853720 10.1038/ejhg.2017.128 PMC5643963

[98] Y Chromosome Loss Drives Bladder Cancer Aggressiveness and Immune Evasion. Cancer Discov. 2023;13(8):1761. 10.1158/2159-8290.CD-RW2023-102. 10.1158/2159-8290.CD-RW2023-102 37387586

[99] Li J, Lan Z, Liao W, et al. Histone demethylase KDM5D upregulation drives sex differences in colon cancer. Nature. 2023;619(7970):632–9. 10.1038/s41586-023-06254-7. 37344599 10.1038/s41586-023-06254-7 PMC10529424

[100] Liu F, Yuan JH, Huang JF, et al. Long noncoding RNA FTX inhibits hepatocellular carcinoma proliferation and metastasis by binding MCM2 and miR-374a. Oncogene. 2016;35(41):5422–34. 10.1038/onc.2016.80. 27065331 10.1038/onc.2016.80

[101] Huang C, Azizi P, Vazirzadeh M, et al. Non-coding RNAs/DNMT3B axis in human cancers: from pathogenesis to clinical significance. J Transl Med. 2023;21:621. 10.1186/s12967-023-04510-y. 37705098 10.1186/s12967-023-04510-y PMC10500757

[102] Hua JT, Chen S, He HH. Landscape of noncoding RNA in prostate cancer. Trends Genet. 2019;35(11):840–51. 10.1016/j.tig.2019.08.004. 31623872 10.1016/j.tig.2019.08.004

[103] Zhu J, Li Q, Wu Z, Xu W, Jiang R. Circular RNA-mediated miRNA sponge & RNA binding protein in biological modulation of breast cancer. Noncoding RNA Res. 2024;9(1):262–76. 10.1016/j.ncrna.2023.12.005. 38282696 10.1016/j.ncrna.2023.12.005 PMC10818160

[104] Fan YJ, Ding Z, Zhang Y, et al. Sex-lethal regulates back-splicing and generation of the sex-differentially expressed circular RNAs. Nucleic Acids Res. 2023;51(10):5228–41. 10.1093/nar/gkad280. 37070178 10.1093/nar/gkad280 PMC10250224

[105] Liu S, Lai W, Shi Y, et al. Annotation and cluster analysis of long noncoding RNA linked to male sex and estrogen in cancers. NPJ Precis Oncol. 2020;4:5. 10.1038/s41698-020-0110-5. 32195358 10.1038/s41698-020-0110-5 PMC7054536

[106] Li J, Ming Z, Yang L, Wang T, Liu G, Ma Q. Long noncoding RNA XIST: mechanisms for X chromosome inactivation, roles in sex-biased diseases, and therapeutic opportunities. Genes Dis. 2022;9(6):1478–92. 10.1016/j.gendis.2022.04.007. 36157489 10.1016/j.gendis.2022.04.007 PMC9485286

[107] Shao T, Xie Y, Shi J, et al. Surveying lncRNA-lncRNA cooperations reveals dominant effect on tumor immunity cross cancers. Commun Biol. 2022;5:1324. 10.1038/s42003-022-04249-0. 36463330 10.1038/s42003-022-04249-0 PMC9719535

[108] Li X, Wu Y, Jin Y. Exosomal lncRNAs and circRNAs in lung cancer: emerging regulators and potential therapeutic targets. Noncoding RNA Res. 2024;9(4):1069–79. 10.1016/j.ncrna.2024.06.010. 39022675 10.1016/j.ncrna.2024.06.010 PMC11254510

[109] Yu SH, Jung SI. The potential role of urinary microbiome in benign prostate hyperplasia/lower urinary tract symptoms. Diagnostics (Basel). 2022;12(8):1862. 10.3390/diagnostics12081862. 36010213 10.3390/diagnostics12081862 PMC9406308

[110] Kim MS, Jung SI. The urinary tract microbiome in male genitourinary diseases: focusing on benign prostate hyperplasia and lower urinary tract symptoms. Int Neurourol J. 2021;25(1):3–11. 10.5213/inj.2040174.087. 33504133 10.5213/inj.2040174.087 PMC8022174

[111] Parra-Grande M, Oré-Arce M, Martínez-Priego L, et al. Profiling the bladder microbiota in patients with bladder cancer. Front Microbiol. 2022;12:718776. 10.3389/fmicb.2021.718776. 35197936 10.3389/fmicb.2021.718776 PMC8859159

[112] Bilski K, Żeber-Lubecka N, Kulecka M, et al. Microbiome sex-related diversity in non-muscle-invasive urothelial bladder cancer. Curr Issues Mol Biol. 2024;46(4):3595–609. 10.3390/cimb46040225. 38666955 10.3390/cimb46040225 PMC11048804

[113] Heidar NA, Bhat TA, Shabir U, Hussein AA. The urinary microbiome and bladder cancer. Life. 2023;13(3):812. 10.3390/life13030812. 36983967 10.3390/life13030812 PMC10053959

[114] Jing N, Wang L, Zhuang H, Ai C, Jiang G, Liu Z. Sex-biased immune responses to antibiotics during anti-PD-L1 treatment in mice with colon cancer. J Immunol Res. 2022;2022:9202491. 10.1155/2022/9202491. 35903754 10.1155/2022/9202491 PMC9325566

[115] Liu F, Han Z, Lu J, Zhong W. Development and validation of a tobacco smoking-related index for predicting overall survival and immunotherapy response in bladder cancer. Environ Sci Pollut Res Int. 2023;30(26):68701–15. 10.1007/s11356-023-27132-9. 37129813 10.1007/s11356-023-27132-9

[116] Xiong J, Yang L, Deng YQ, et al. The causal association between smoking, alcohol consumption and risk of bladder cancer: A univariable and multivariable Mendelian randomization study. Int J Cancer. 2022;151(12):2136–43. 10.1002/ijc.34228. 35904850 10.1002/ijc.34228

[117] Doshi B, Athans SR, Woloszynska A. Biological differences underlying sex and gender disparities in bladder cancer: current synopsis and future directions. Oncogenesis. 2023;12(1):44. 10.1038/s41389-023-00489-9. 37666817 10.1038/s41389-023-00489-9 PMC10477245

[118] Shala NK, Stenehjem JS, Babigumira R, et al. Exposure to benzene and other hydrocarbons and risk of bladder cancer among male offshore petroleum workers. Br J Cancer. 2023;129(5):838–51. 10.1038/s41416-023-02357-0. 37464024 10.1038/s41416-023-02357-0 PMC10449774

[119] Hosseini B, Zendehdel K, Bouaoun L, et al. Bladder cancer risk in relation to occupations held in a nationwide case-control study in Iran. Int J Cancer. 2023;153(4):765–74. 10.1002/ijc.34560. 37158123 10.1002/ijc.34560

[120] Masaoka H, Matsuo K, Sawada N, et al. Alcohol consumption and bladder cancer risk with or without the flushing response: The Japan Public Health Center-based Prospective Study. Int J Cancer. 2017;141(12):2480–8. 10.1002/ijc.31028. 28875523 10.1002/ijc.31028

[121] Botteri E, Ferrari P, Roswall N, et al. Alcohol consumption and risk of urothelial cell bladder cancer in the European prospective investigation into cancer and nutrition cohort. Int J Cancer. 2017;141(10):1963–70. 10.1002/ijc.30894. 28722206 10.1002/ijc.30894

[122] Mahamat-Saleh Y, Al‐Rahmoun M, Severi G, et al. Baseline and lifetime alcohol consumption and risk of skin cancer in the European Prospective Investigation into Cancer and Nutrition cohort (EPIC). Int J Cancer. 2023;152(3):348–62. 10.1002/ijc.34253. 36053839 10.1002/ijc.34253 PMC10087036

[123] Antwi SO, Eckel-Passow JE, Diehl ND, et al. Alcohol consumption, variability in alcohol dehydrogenase genes and risk of renal cell carcinoma. Int J Cancer. 2018;142(4):747–56. 10.1002/ijc.31103. 29023769 10.1002/ijc.31103

[124] Yu EYW, Wesselius A, Mehrkanoon S, et al. Vegetable intake and the risk of bladder cancer in the BLadder cancer epidemiology and nutritional determinants (BLEND) international study. BMC Med. 2021;19:56. 10.1186/s12916-021-01931-8. 33685459 10.1186/s12916-021-01931-8 PMC7942172

[125] Dianatinasab M, Wesselius A, Salehi-Abargouei A, et al. Adherence to a western dietary pattern and risk of bladder cancer: a pooled analysis of 13 cohort studies of the Bladder cancer epidemiology and nutritional determinants international study. Int J Cancer. 2020;147(12):3394–403. 10.1002/ijc.33173. 32580241 10.1002/ijc.33173 PMC7689707

[126] Jochems SHJ, Reulen RC, van Osch FHM, et al. Fruit consumption and the risk of bladder cancer: a pooled analysis by the Bladder cancer epidemiology and nutritional determinants study. Int J Cancer. 2020;147(8):2091–100. 10.1002/ijc.33008. 32285440 10.1002/ijc.33008

[127] Choi MG, Choi CM, Lee DH, et al. Impact of gender on response to immune checkpoint inhibitors in patients with non-small cell lung cancer undergoing second- or later-line treatment. Transl Lung Cancer Res. 2022;11(9):1866–76. 10.21037/tlcr-22-146. 36248340 10.21037/tlcr-22-146 PMC9554675

[128] Klein SL, Flanagan KL. Sex differences in immune responses. Nat Rev Immunol. 2016;16(10):626–38. 10.1038/nri.2016.90. 27546235 10.1038/nri.2016.90

[129] Vavalà T. Immunotherapy outcomes in non-small cell lung cancer according to a gender perspective. Prog Mol Biol Transl Sci. 2024;209:241–58. 10.1016/bs.pmbts.2024.09.004. 39461754 10.1016/bs.pmbts.2024.09.004

[130] Singh S, Singh N, Baranwal M, Sharma S, Devi SSK, Kumar S. Understanding immune checkpoints and PD-1/PD-L1-mediated immune resistance towards tumour immunotherapy. 3 Biotech. 2023;13(12):411. 10.1007/s13205-023-03826-2. 37997595 10.1007/s13205-023-03826-2 PMC10663421

[131] Ashrafizadeh M, Zarrabi A, Hushmandi K, et al. PD-1/PD-L1 axis regulation in cancer therapy: the role of long non-coding RNAs and microRNAs. Life Sci. 2020;256:117899. 10.1016/j.lfs.2020.117899. 32504749 10.1016/j.lfs.2020.117899

[132] Conforti F, Pala L, Pagan E, et al. Sex-based differences in response to anti-PD-1 or PD-L1 treatment in patients with non-small-cell lung cancer expressing high PD-L1 levels. A systematic review and meta-analysis of randomized clinical trials. ESMO Open. 2021;6(5):100251. 10.1016/j.esmoop.2021.100251. 34455288 10.1016/j.esmoop.2021.100251 PMC8403740

[133] Conforti F, Pala L, Bagnardi V, et al. Cancer immunotherapy efficacy and patients’ sex: a systematic review and meta-analysis. Lancet Oncol. 2018;19(6):737–46. 10.1016/S1470-2045(18)30261-4. 29778737 10.1016/S1470-2045(18)30261-4

[134] Bellmunt J, Wit R, Vaughn DJ, et al. Pembrolizumab as second-line therapy for advanced urothelial carcinoma. N Engl J Med. 2017;376(11):1015. 10.1056/NEJMoa1613683. 28212060 10.1056/NEJMoa1613683 PMC5635424

[135] Balar AV, Galsky MD, Rosenberg JE, et al. Atezolizumab as first-line treatment in cisplatin-ineligible patients with locally advanced and metastatic urothelial carcinoma: a single-arm, multicentre, phase 2 trial. Lancet. 2017;389(10064):67–76. 10.1016/S0140-6736(16)32455-2. 27939400 10.1016/S0140-6736(16)32455-2 PMC5568632

[136] Tao X, Wang Y, Xiang B, et al. Sex bias in tumor immunity: insights from immune cells. Theranostics. 2025;15(11):5045–72. 10.7150/thno.106465. 40303343 10.7150/thno.106465 PMC12036885

[137] Bader DA, Chakraborty B, McDonnell DP, Hirschey MD. Targeting androgen receptor signaling to enhance cancer immunotherapy. Trends Pharmacol Sci. 2025. 10.1016/j.tips.2025.11.003. 41344935 10.1016/j.tips.2025.11.003 PMC12708014

[138] X Z, L C, C G, et al. Androgen signaling contributes to sex differences in cancer by inhibiting NF-κB activation in T cells and suppressing antitumor immunity. Cancer Res. 2023. 10.1158/0008-5472.CAN-22-2405. 10.1158/0008-5472.CAN-22-2405 36634207

[139] Wu W, Maneix L, Insunza J, et al. Estrogen receptor β, a regulator of androgen receptor signaling in the mouse ventral prostate. Proc Natl Acad Sci U S A. 2017;114(19):E3816–22. 10.1073/pnas.1702211114. 28439009 10.1073/pnas.1702211114 PMC5441728

[140] Pinton G, Nilsson S, Moro L. Targeting estrogen receptor beta (ERβ) for treatment of ovarian cancer: importance of KDM6B and SIRT1 for ERβ expression and functionality. Oncogenesis. 2018;7(2):15. 10.1038/s41389-018-0027-9. 29422491 10.1038/s41389-018-0027-9 PMC5833712

[141] Ou Z, Wang Y, Chen J, et al. Estrogen receptor β promotes bladder cancer growth and invasion via alteration of miR-92a/DAB2IP signals. Exp Mol Med. 2018;50(11): 152. 10.1038/s12276-018-0155-5. 30459405 10.1038/s12276-018-0155-5 PMC6243995

[142] Tong D. Selective estrogen receptor modulators contribute to prostate cancer treatment by regulating the tumor immune microenvironment. J Immunother Cancer. 2022;10(4):e002944. 10.1136/jitc-2021-002944. 35383112 10.1136/jitc-2021-002944 PMC8984050

[143] Goto T, Kashiwagi E, Jiang G, et al. Estrogen receptor-β signaling induces cisplatin resistance in bladder cancer. Am J Cancer Res. 2020;10(8):2523–34. 32905529 PMC7471368

[144] Zhang CM, Ge ZB, Zhou HH, et al. Sex chromosomes/hormones and the tumor microenvironment of non-reproductive cancers. Front Immunol. 2025;16:1642956. 10.3389/fimmu.2025.1642956. 41019050 10.3389/fimmu.2025.1642956 PMC12463827

[145] Sanchez LAC, Rheinbay E. Lost but not least: Y chromosome loss as a driver of cancer. Trends Cancer. 2025. 10.1016/j.trecan.2025.11.009. 10.1016/j.trecan.2025.11.009 41390309

[146] Hurst CD, Alder O, Platt FM, et al. Genomic Subtypes of Non-Invasive Bladder Cancer with Distinct Metabolic Profile, Clinical Outcome and Female Gender Bias in KDM6A Mutation Frequency. Cancer Cell. 2017;32(5):701–e7157. 10.1016/j.ccell.2017.08.005. 29136510 10.1016/j.ccell.2017.08.005 PMC5774674

[147] Zhang J, Xiang S, Liu D, et al. KDM6A downregulation promotes tumor-prone cytokines expression in cancer-associated fibroblasts by activating enhancers. Cell Death Dis. 2025;16(1):523. 10.1038/s41419-025-07818-3. 40659611 10.1038/s41419-025-07818-3 PMC12259948

[148] Rubin JB, Lagas JS, Broestl L, et al. Sex differences in cancer mechanisms. Biol Sex Differ. 2020;11:17. 10.1186/s13293-020-00291-x. 32295632 10.1186/s13293-020-00291-x PMC7161126

[149] Yuan Y, Liu L, Chen H, et al. Comprehensive characterization of molecular differences in cancer between male and female patients. Cancer Cell. 2016;29(5):711–22. 10.1016/j.ccell.2016.04.001. 27165743 10.1016/j.ccell.2016.04.001 PMC4864951

