# Supplementary material for: Molecular mechanisms and clinical impacts of sex differences in urologic cancers
Source: Discov Oncol. 2026 May 3;17:930. doi: 10.1007/s12672-026-05029-6 (PMC13283953; doi:10.1007/s12672-026-05029-6)
Supplement: Supplementary file 1 — Supplementary Material 1 [file 12672_2026_5029_MOESM1_ESM.docx]

**Molecular Mechanisms and Clinical Impacts of Sex Differences in Urologic Cancers**

**Pengcheng Fu^1#^, Dong Wen^1#^, Geliu Shuai^1^, Yuebin Wang^1^, Shengxin Yu^1^, Yuanri Xie^1^, Pufang Xue^1^,Zhenqin Liang^1^, Junrong Zou^1-5^,**

**Xiaofeng Zou^1-5*^**

**Pengcheng Fu ORCID:** [**https://orcid.org/0009-0003-7850-5308**](https://orcid.org/0009-0003-7850-5308)

**Junrong Zou ORCID:** [**https://orcid.org/0000-0003-4409-5294**](https://orcid.org/0000-0003-4409-5294)

***Correspondence:**

**Xiaofeng Zou**

**Email：**[**gyfyzouxf@126.com**](mailto:gyfyzouxf@126.com)

**ORCID:**[**https://orcid.org/0000-0002-7472-4488**](https://orcid.org/0000-0002-7472-4488)

1. The First Clinical College, Gannan Medical University, Ganzhou, Jiangxi, China.

2. Department of Urology, The First Affiliated hospital of Gannan Medical University; Ganzhou, Jiangxi, China 340001

3. Institute of Urology, The First Affiliated hospital of Gannan Medical University, Ganzhou, Jiangxi, China 340001

4. Institute of Hematological Research, Shaanxi Provincial People’s Hospital, 256 West Youyi Road, Xi’an, Shaanxi China 71000

5. Ganzhou Key Laboratory of Rare Earth Materials and Human Health, Ganzhou, Jiangxi, China 340001

# Abstract:

Urological cancers exhibit significant sex differences in incidence, treatment response, and prognosis, with males generally showing higher morbidity and mortality. This review systematically summarizes the underlying molecular and clinical mechanisms of these disparities, focusing on sex hormones, chromosome biology, tumor immune microenvironment, and microbiota. Sex hormones modulate key tumor processes including proliferation, apoptosis, non-apoptotic cell death, and DNA repair. Genetic factors such as X chromosome inactivation escape genes and Y chromosome loss also contribute to sex-biased cancer susceptibility. Furthermore, sex-specific differences in the urinary system and gut microbiota influence local immunity and inflammation, thereby affecting tumor progression and therapeutic response. Lifestyle and environmental factors, including smoking, alcohol consumption, and occupational exposures, further exacerbate these disparities. Clinically, sex differences impact the efficacy of immunotherapy and targeted therapies, underscoring the need for sex-informed treatment strategies. Integrating sex as a biological variable in research, clinical practice, and public health policies is essential for advancing precision oncology in urologic cancers.**Keywords:** Urologic Neoplasms; sex bias; sex hormone; bladder cancer; androgen receptors

# Introduction：Urologic cancers exhibit significant sex disparities, with men showing higher incidence and mortality in malignancies such as bladder and kidney cancer[1,2] (Fig.1). These differences are influenced not only by lifestyle and environmental factors but also by intrinsic biological mechanisms. This review aims to synthesize current evidence on the biological basis of sex differences in urologic cancers, focusing on sex hormones[3], chromosome effects[4], immune microenvironment[5], and microbiota[6]. A systematic literature search was conducted using PubMed, Web of Science, and Scopus, with keywords including "urologic neoplasms," "sex bias," "sex hormones," and "immunotherapy." Evidence indicates that sex hormones and their receptors regulate key tumor processes including proliferation[7], apoptosis[8], and DNA repair[9]. Sex chromosome genes, especially those escaping X-inactivation [4]and Y chromosome genes[10], also contribute to cancer risk. Additionally, sex-specific differences in urinary[11] and gut[6] microbiota influence local immunity and inflammation, further modulating cancer development and treatment response. Despite growing knowledge, the integration of sex-specific factors into research and clinical practice remains inadequate. This gap has direct clinical consequences. Understanding sex differences is critical for optimizing treatment selection predicting patient response, developing sex-specific biomarkers, and designing balanced clinical trials. This review highlights these mechanisms and discusses their implications for developing sex-informed therapeutic strategies, ultimately supporting the advancement of precision medicine in urologic oncology.(Fig. 2)

# 1. Hormonal Mechanisms in Urologic Cancers

## 1.1 Regulation of physiological processes by Sex Hormones

### 1.1.1 Sex hormones and Cell Proliferation

Sex hormones regulate cell proliferation by binding to nuclear receptor or by influencing other signaling pathways. In the pro-cancer mechanism, estrogen induces the expression of Cyclin D1 by activating the Wnt/β-catenin pathway[12], accelerates the G1/S phase transition, and promotes cells to enter the S phase **(Fig.3)**[7]. However, its effects are tissue-specific; in uterine leiomyomas, estrogen enhances vitamin D3 synthesis and activation, which inhibits proliferation via vitamin D receptor signaling [13][14,15].

Androgens downregulate TGF-β receptor I (TβRI) expression through Androgen receptor（AR）, attenuating the tumor suppressive function of TGF-β **(Fig.3)**[16–18]. After the inactivation of TGF-β signaling, its original proliferative inhibition and pro-apoptotic effects are relieved, and the migration and invasion of tumor cells are promoted instead[18]. Androgen inhibition of the TGF-β signaling pathway may also further promote tumor initiation and progression by affecting immune cell function in the tumor microenvironment[19] Dihydrotestosterone (DHT) can also promote BCa proliferation and invasion via a non-AR-dependent pathway by binding to Epiplakin1 (EPPK1), which activates the p38 MAPK/c-Jun axis and upregulates Junction Plakoglobin (JUP) expression [20]. Clinical data have shown that high JUP expression is significantly associated with poor prognosis in patients, and blocking this pathway (e.g., knockdown of EPPK1) is effective in slowing tumor progression[20,21]. This mechanism reveals the critical role of the EPPK1-mediated MAPK/JUP signaling axis in BCa[22], providing a new strategy for targeted therapy.

### 1.1.2 Sex hormones and apoptosis

Sex hormones regulate apoptosis, influencing urological tumor progression. Estrogen can inhibit apoptosis by suppressing MAPK signaling pathway activity [3]. Estrogen can inhibit the expression of Fas and FasL, thereby reducing the occurrence of apoptosis[25]. Estrogen can also influence apoptosis by modulating the activity of key proteins in the death receptor signaling pathway, such as caspase-8[25].

Androgens enhance the proliferation, invasion, and anti-apoptotic capacity of BCa cells through AR[20]. For example, dihydrotestosterone can promote tumor progression by activating AR signaling, upregulating oncogenesand inhibiting the expression of apoptosis-related proteins[22]. In male-specific prostate cancer, androgen/AR signaling drives tumor progression by activating growth factor pathways, promoting cell proliferation and inhibiting apoptosis in prostate cancer [26,27], and AR signaling also maintains tumor cell survival by regulating cell cycle-related genes (e.g., p21, p15, p16) and DNA repair mechanisms[28]. The above conclusions suggest that sex hormones play a complex role in a variety of signaling pathways, thereby finely regulating apoptosis.

1.1.3 Sex Hormones and Non-Apoptotic Cell Death

In addition to classical apoptosis regulation, sex hormones are also involved in regulating a variety of non-apoptotic cell death pathways, including ferroptosis[27]、pyroptosis[28],and autophagy[28].These mechanisms contribute to sex differences in urinary cancer development.

Ferroptosis is an iron-dependent form of regulated cell death driven by lipid peroxidation, the occurrence of which is closely associated with cellular metabolic status and levels of oxidative stress[29]. In gynecological cancers, estrogen's antioxidant properties can suppress ferroptosis by upregulating GPX4, a mechanism that may be relevant in hormone-responsive urologic tumors.[29,30]. A clear interaction exists between AR signaling pathways and ferroptosis[31]. Antiandrogen agents, when combined with iron-containing compounds, can enhance ferroptosis by promoting oxidative damage. Meanwhile, androgen deprivation therapy may influence cellular susceptibility to ferroptosis through alterations in the expression of iron metabolism-related genes[32]. In addition, the PI3K–AKT–mTOR signaling pathway is also involved in the regulation of ferroptosis. In prostate cancer, this pathway is modulated by androgen signaling and can suppress lipid peroxidation via the SREBP1/SCD1 axis, thereby conferring resistance to ferroptosis[33].

Pyroptosis is an inflammatory mode of programmed cell death mediated by gasdermin proteins, characterized by the extensive release of inflammatory factors that significantly influence the immune status of the tumor microenvironment [34,35]. It has dual roles: inducing anti-tumor immunity or, if excessive, promoting immunosuppression[36]. The inflammation-dependent mechanism of pyroptosis provides a theoretical basis for its potential regulation. Future studies should explore the interaction of sex hormones with these pathways to elucidate their specific roles in tumor immunity.

As a crucial intrinsic self‑regulatory mechanism, the interplay between autophagy and sex hormones in tumorigenesis is spatiotemporally dynamic and tissue‑specific[37]. Estrogen exerts bidirectional regulation via the autophagy pathway: early activation of cytoprotective autophagy serves to restrain tumor initiation, whereas in advanced stages, autophagy is co‑opted to facilitate tumor adaptation and survival[38]. In contrast, androgens predominantly display unidirectional regulatory features that promote tumor progression[39]. Gender differences markedly influence basal autophagic activity as well as tumor susceptibility, and such interactions are intimately linked to clinical therapeutic resistance [40,41]. Targeting the “sex hormone–autophagy” regulatory axis holds promise as a novel therapeutic strategy for hormone‑dependent tumors exhibiting gender‑related sensitivity.

### 1.1.4 Sex hormones and DNA repair mechanisms

Estrogen signaling is known to modulate DNA repair pathways, influencing therapy resistance in hormone-responsive cancers. In breast cancer models, estrogen via ERα enhances repair pathways like homologous recombination and base excision repair, contributing to radio- and chemoresistance [9,29,30]. Whether similar estrogen-driven DNA repair mechanisms operate in urologic cancers like BCa remains largely unexplored and represents a key knowledge gap.Among them, the ER signal activates the Base Excision Repair (BER) pathway, which regulates the activity of repair enzymes such as OGG1 to remove oxidative damage products such as 8-oxygen guanine[31]. Disparities and Trends. However, in the obesity-related microenvironment, estrogen synergizes with leptin, insulin, and other factors to increase the accumulation of DNA damage and form a cancer-promoting environment[32].In epigenetic regulation, the ER recruits epiregulators such as histone demethylase (KDM4B) to remodel chromatin accessibility at sites of DNA damage[33,34]. This regulation may both enhance the efficiency of repair in breast cancer and may lead to an increase in the rate of repair errors.

In prostate cancer, AR directly upregulates CRY1, a transcriptional coregulator that enhances the efficiency of non-homologous end joining (NHEJ) DNA repair. High CRY1 expression is linked to poorer patient prognosis.[35]. AR does not directly regulate the transcription of DNA damage response genes[36],and AR signaling upregulates the expression of DNA damage response genes such as ATM and RAD51, such as in prostate cancer cells carried by the HSD3B1 (1245C) allele, AR activation leads to enhanced expression of genes related to DNA double-strand break repair, accelerates DNA repair and promotes radiotherapy resistance[37]. Poly ADP-ribose Polymerase inhibitors, which target DNA damage repair mechanisms, exhibit particular efficacy in Homologous Recombination (HR)-deficient prostate cancer [38].

## 1.2. Expression Patterns and Functional Roles of Sex Hormone Receptors in Urologic Cancers

1.2.1 Dual role of the estrogen receptor

Estrogen receptor α (ERα) and β (ERβ) are the two main estrogen receptor subtypes, and they are known to differ significantly in structure and function. In BCa tissues, the mRNA and protein expression levels of ERα were significantly higher than those in normal tissues, especially in immortalized urothelial cells and bladder tumor cells, while the expression levels of ERβ were nearly identical in the two tissues[39].However, a small number of immunohistochemical studies have found that the expression of ERα in BCa tissues may be reduced compared with non-tumor tissues, suggesting that the expression of ERα may be affected by the tumor microenvironment or detection methods[40].In general, the effect of ERα may promote tumor proliferation, etc., while the expression of ERβ is not significantly different, and there may be anti-tumor effects.

ERα can also promote cell cycle progression and tumor growth by activating signaling pathways such as MAPK/ERK and PI3K/AKT[3].ERα expression is upregulated in malignant transformed prostate epithelial cells and increased in malignant and metastatic prostate cancers, and ERα signaling can also promote tumor cell invasion by regulating the degradation of the extracellular matrix and cell motility[42].

ERβ is mainly expressed in epithelial cells, has protective and anti-apoptotic effects, and is able to inhibit the proliferation and migration of tumor cells. ERβ can downregulate the expression of CyclinD1 and prevent cells from moving from G1 phase to S phase, thereby slowing down the rate of cell proliferation, while ERβ can also upregulate some genes that inhibit cell proliferation, such as p21 and p27, to further inhibit cell proliferation[41,43]. ERβ is cross-regulated with AR, directly or indirectly influencing AR expression and inducing apoptosis and inhibiting tumor progression by competing with AR to bind to or regulating the expression and activity of intracellular transcription factors[44]. It is important to note that different splice variants of ERβ may have different functions, and in breast cancer, splice variants such as ERβ2 and ERβ5 are associated with high Ki-67 positivity, poor prognostic markers, and promotion of cell migration and invasion[45]. Whether this applies to urinary tract tumors requires further investigation. However, the lack of specificity of existing antibodies leads to contradictory results in the study of splice variants, and further experiments are needed to verify their function.

The opposing functions of ERα and ERβ in urologic cancers are largely attributed to their distinct cellular distributions: ERα predominates in stromal/mesenchymal cells, often associating with pro-tumorigenic pathways, while ERβ is mainly expressed in epithelial cells and frequently correlates with anti-proliferative effects[41,43]. This functional dichotomy, however, is not absolute and can be modulated by factors like androgen levels, which may suppress ERβ activity and tilt the balance toward ERα-driven oncogenesis [46]. Critically, the net effect of estrogen signaling in a given tumor likely depends on the ERα:ERβ expression ratio, receptor splice variants, and crosstalk with other pathways[60,61]. Targeting this regulatory axis with selective estrogen receptor modulators (SERMs) represents a rational therapeutic strategy to exploit this functional conflict [47].

**1.2.2 AR Expression in Bladder Cancer Progression**

AR is sex-significantly expressed in BCa, and AR expression levels are higher in men than in women, which may partly explain the higher prevalence of BCa in men than in women[22]. However, AR expression levels are inversely correlated with tumor stage and grade: AR expression is high in low-grade (Ta-T1) non-muscle-invasive bladder cancer (NMIBC), but significantly decreases in high-grade or muscle-invasive bladder cancer (MIBC)[48,49]. Changes in the expression of AR dynamics suggest that it may have a pro-tumor effect in the early stages of BCa, but its expression is gradually suppressed as the tumor progresses, which may be associated with tumor dedifferentiation or metastasis[50,51].

## 1.3 Immune Modulation by Sex Hormones

### 1.3.1 Neutrophils

Estrogen reprograms neutrophil gene expression and function. In preclinical models, estrogen-activated neutrophils release pro-inflammatory factors like IL-1β and IL-8, fostering a pro-tumorigenic microenvironment.[52].

We know that the high incidence of bladder cancer is associated with androgen levels in men Androgens suppress neutrophil cytotoxicity by upregulating TGF-β receptor I (TβRI). This suppression can be reversed by either high-dose testosterone or TβRI inhibitors, restoring anti-tumor activity. [19].Programmed cell death ligand 1 (PD-L1) expressed by tumor cells blocks neutrophil cytotoxicity, while androgens may further inhibit its antitumor effects by enhancing PD-L1 expression[19,56].Androgen receptor signaling can also block T cell function by upregulating USP18 by upregulating NF-κB activity[57], indirectly affecting neutrophil function and forming an immunosuppressive tumor microenvironment. Laura Pala et al. showed that androgen deprivation therapy (ADT) enhanced the efficacy of anti-PD-1 (Programmed cell death protein 1) immunotherapy[58],indirectly reflecting the inhibition of T cell function by AR signaling.

### 1.3.2 Macrophage

Classical models divide macrophages into two polarized states: M1 (pro-inflammatory anti-tumor) and M2 (anti-inflammatory pro-tumor)[59]. The M1 macrophages mediate cytotoxicity via mechanisms like ADCC and T/NK cell activation, whereas M2 macrophages promote immunosuppression, angiogenesis, and metastasis through secretion of factors such as IL-10 and Vascular Endothelial Growth Factor (VEGF).[60].This binary classification is particularly important in the tumor microenvironment, as M2 macrophages are often associated with immune evasion[61].

E2 can modulate the NF-κB signaling pathway by enhancing IκBα levels through ERα and influencing p65 binding at inflammatory gene promoters [61] Estrogen also signals through the non-canonical receptor G Protein-Coupled Estrogen Receptor 1(GPER1) to inhibit macrophage proliferation and PD-L1 expression, primarily by suppressing the MEK/ERK/cyclin D1 pathway.[62]..Estrogen also inhibits IL-1β production by binding to estrogen receptors (ERs) in neutrophils and macrophages, and reduces its pro-inflammatory effects[63]. These mechanisms lead to enhanced anti-inflammatory properties of M2-type macrophages. Barcena et al. noted that estrogen inhibits the pro-inflammatory phenotype of macrophages, namely M1 type,by upregulating Sirt3 expression[64]. In urologic tumors, the enrichment of M2-type macrophages is associated with tumor metastasis and the immunosuppressive microenvironment[65]. The regulation of estrogen on macrophages needs to be further explored.

Androgens promote macrophage polarization to M2 type by regulating the expression of local cytokines, such as CXCL1 and G-CSF[66]. M2 macrophages form a pro-tumor microenvironment by secreting anti-inflammatory factors (e.g., IL-10) and inhibiting T cell activity[67]. Macrophages are rich in cholesterol and capable of transferring cholesterol into tumor cells[67,68]. Macrophages promote androgen synthesis by enhancing cholesterol uptake by tumor cells, thereby activating AR signaling in tumor cells[68].In addition, AR is also expressed in macrophages, and by activating the AR signaling pathway, macrophage-like THP-1 cell lines can upregulate the expression ofTriggering Receptor Expressed on Myeloid cells-1 and its downstream cytokines, thereby promoting the migration and invasion of certain tumors such as PCa cells[69]. Androgens not only directly inhibit the bactericidal function of neutrophils (e.g., reduced degranulation and phagocytic activity), but may also indirectly affect the function of other immune cells (e.g., T cells, NK cells) through macrophages[70]. In BCa, androgen-AR signaling not only promotes tumor invasion, but may also recruit immune cells into the tumor microenvironment to form a vicious cycle[22]. This may be one of the important reasons for the significant sex difference in the incidence of BCa.

### 1.3.3 T cells

Estrogen bidirectionally modulates T cell immunity: low levels promote T Helper 1 Cell (Th1) responses, while high levels favor T Helper 2 Cell (Th2) polarization.[3,61]. The regulation of CD8+ T cells by estrogen is bidirectional. On the one hand, estrogen activates the TCR signaling of CD8+ T cells through ERβ and enhances their anti-tumor function. On the other hand, estrogen inhibits the activity of CD8+ T cells by promoting the expansion and function of regulatory T cells (Tregs), thereby weakening the anti-tumor immune response[3,71].In urinary tract tumors, lower androgen levels in women and the protective effects of estrogen may work together to reduce tumor aggressiveness[72,73].

Androgens act directly within CD8+ T cells via AR signaling to regulate Tcf7, a master regulator of T cell differentiation. This drives CD8+ T cells toward an exhausted phenotype (characterized by high PD-1 expression) and impairs their anti-tumor function.[5], which is manifested by reduced cytotoxicity and reduced stem-cell-like properties[74]. This inhibitory effect is more pronounced in men, leading directly to accelerated progression of urologic tumors such as BCa and poor response to immunotherapy[75]. Androgen deprivation (e.g., surgical castration or treatment with the small molecule inhibitor abiraterone) has been shown to restore the anti-tumor activity of CD8+ T cells, thereby enhancing the efficacy of PD-1 inhibitors[22,76].In addition, the higher ratio of M2 macrophages to Treg in the human male tumor microenvironment indirectly inhibits CD8+ T cell function[62,77]. This may be one of the important factors in the sex difference in tumor incidence.

# **Limitation and Translational Consideration:** Several mechanisms discussed in this review are informed by studies in non-urologic cancers. While these models provide valuable hypotheses, direct translation to bladder or renal cancers requires validation. We have prioritized evidence from urologic malignancies where available and indicate when mechanisms are proposed based on conserved pathways. Furthermore, while we have focused on the most established roles of estrogens and androgens, the potential influence of other hormonal systems (e.g., progesterone, glucocorticoids) on the tumor–stromal interface represents an important area for future research to fully delineate the endocrine landscape of sex disparities.2. Genetic and Epigenetic Contributions

## 2.1 X Chromosome Inactivation and Escape Genes in Cancer Susceptibility

In female mammals, X chromosome inactivation (XCI) randomly silences one of the two X chromosomes to achieve dose compensation[78]. Notably, partially evading X-inactivated tumor suppressors (EXITS) such as ATRX, CNKSR2, DDX3X, KDM5C, KDM6A, and MAGEC3 remain active on both X chromosomes, thereby reducing cancer risk in women to some extent[79,80]. This phenomenon suggests that the XCI escape gene plays a non-negligible role in women's cancer risk. Because there is only one X chromosome in males, a mutation can cause a complete loss of gene function[81]. However, women need two alleles to be inactivated at the same time to be inactivated, which relatively reduces the risk of tumorigenesis. Xiaoxi Li et al. observed a significant increase in tumor incidence due to reduced UTX copy number in a mouse lymphoma model, with a higher incidence of lymphoma in female mice with a single copy of UTX than in double copies, and the highest incidence of tumors in male mice with complete UTX knockout[82].This negative correlation of gene dose with tumorigenesis can be used as direct evidence to support that UTX is one of the EXITS genes. In women, XCI maintains the biallelic expression of tumor suppressor genes such as KDM6A, forming a double protective effect on cancer. This sex difference not only explains the higher cancer susceptibility in men but also provides a molecular basis for the development of sex-specific precision treatment strategies.

## 2.2 Y Chromosome Loss and Its Oncogenic Impact

Y chromosome deletion (LOY) is the most common somatic mutation in male individuals[83]. The biological mechanism of Y chromosome deletion and tumorigenesis deletion or dysfunction of Y chromosome genes, can affect tumorigenesis through a variety of pathways. The incidence of mLOY (mosaic Y chromosome deletion) increases significantly with age (approximately 1.6-fold every 10 years)[84]. Large-scale cohort studies have shown that mLOY carriers have an increased risk of developing solid tumors such as lung cancer and BCa, which may be associated with a chronic inflammatory state due to Y gene deletion[85,86]. When the Y chromosome is missing, X chromosome homologous genes may emerge, compensated for high expression, but this compensation may be exploited by tumor cells. For example, prostate cancer cells rely on the homologous gene EIF1AX (Eukaryotic Translation Initiation Factor 1A, X) on the X chromosome to maintain proliferation after the deletion of the Y chromosome, forming a vulnerable target for treatment[87].

Certain genes on the Y chromosome play an important role in regulating the immune response. For example, the UTY (Ubiquitously Transcribed Tetratricopeptide Repeat Gene on Y Chromosome) gene can affect the anti-tumor immune response by regulating macrophage function, and its deletion may lead to weakened macrophage immune surveillance and increased tumor susceptibility[88].Y chromosome deletion (LOY) in BCa is directly related to T cell dysfunction, leading to an immunosuppressive state in the tumor microenvironment that enhances the aggressiveness of BCa[89].Y chromosome genomic protein demethylase KDM5D is a transcriptional upregulated gene driven by KRAS-mediated activation of the STAT4 transcription factor, and its deletion in the mouse CRC (colon cancer) model can cause abnormal chromatin modification, leading to down-regulation of the expression of tight junction-related genes between epithelial cells, and promoting the invasion and metastasis of tumor cells[90].

## 2.3 Non-Coding RNAs as Sex-Specific Regulators

XIST (X-inactive specific transcript) acts as a key regulator of XCI through epigenetic modification mechanisms [91]. In hepatocellular carcinoma, the incidence is significantly higher in males than in females, and studies have found that the high expression of the XIST regulator lnc-FTX in females may inhibit tumor progression by stabilizing XIST, while the lack of XIST in males leads to dysregulation of related tumor suppressor genes[92]. This suggests that aberrant expression of XIST may affect sex differences in tumorigenesis. Dysregulation of XIST may also affect tumor prognosis and staging by regulating the expression of tumor-associated genes on the X chromosome[93].

Long Non-Coding RNAs (LncRNAs) may mediate the regulation of tumor genes by sex hormones, and lncRNAs affect tumor progression by regulating the AR signaling pathway in prostate cancer[94]. circRNAs regulate mRNA stability or translational efficiency, and in breast cancer, circRNAs can affect the splicing and expression of tumor-associated genes by binding to RNA-binding proteins (RBPs)[95]. Some circRNAs may also generate sex-specific cyclic isoforms through reverse splicing, affecting the expression pattern of tumor genes[96].lncRNAs and circRNAs exhibited sex-specific expression patterns in tumors with significant sex differences[97]. The sex-specific expression patterns, stability, and functional relevance of these RNAs make them promising diagnostic and prognostic biomarker[98–100].

# 3. Microbiome and Environmental Influences

## 3.1 Sex-Specific Urinary Microbiota and Bladder Cancer

Under healthy conditions, significant differences exist in the urinary microbiota composition between males and females. A well-established association has been demonstrated between the female urinary tract microbiota and lower urinary tract symptoms (LUTS), whereas no comparable causal relationship has been confirmed in the male population[101,102]. Gender disparities in bladder cancer incidence may be attributed not only to variations in hormone levels, genetic predisposition, and behavioral factors, but also to sex-specific distributions of the genitourinary microbiota, potentially constituting an underlying mechanism. Specifically, enrichment of *Actinobacteria* in female urine samples is significantly associated with lower tumor incidence[103], while males exhibit greater microbial diversity and a higher relative abundance of potential pathogens[104].In patients with NMIBC, a significant correlation is observed between well-differentiated tumor tissue and microbiota diversity, a phenomenon particularly pronounced within the female patient cohort. Notably, these gender-dependent microbial differences are most prominent in the microenvironment of well-differentiated tumors. Female cancer tissues demonstrate a significant increase in the abundance of pro-inflammatory bacterial genera such as Salmonella and Enterobacter, whereas male tissues are characterized by enrichment of opportunistic pathogens including Pseudomona and Acinetobacter[104].Collectively, these findings suggest that the urinary microbiota may influence bladder cancer tumorigenesis and disease progression through sex-specific immunomodulatory mechanisms and inflammatory cascades[105]. It is important to note that most current evidence is correlative, and a direct causal link between specific microbial profiles and sex-biased cancer incidence remains to be conclusively established.

## 3.2 Gut Microbiota Modulates Sex-Biased Anti-Tumor Immunity

In female mouse models, the gut microbiota is characterized by a predominance of Lachnospiraceae. Under estrogen influence, this microbial profile maintains Th17 cell levels, thereby enhancing the anti-tumor efficacy of immune checkpoint inhibitors (ICIs). Conversely, in male mouse models, the gut microbiota is dominated by Muribaculaceae. Testosterone suppresses Th17 cell differentiation and impairs their immunosuppressive function, which conversely cooperatively enhances the anti-tumor activity of CD8⁺ T cells[6,106]. Despite exhibiting distinct gut microbiota compositions, male and female mouse models respectively demonstrate anti-tumor immunity through the specific regulation of Th17 and CD8⁺ T cell functions.

# 4. Lifestyle and environmental factors cannot be ignored

## 4.1 Smoking and chemical exposure

In human society, the prevalence of smoking in men is significantly higher than that in women, and this difference has an important impact on the risk of sex bias in BCa, and smoking is known to be the most well-established risk factor for BCa, with about 50% of BCa cases attributable to smoking[107]. The daily cigarette volume and cumulative smoking index of male smokers were higher than those of women, resulting in the long-term accumulation of carcinogens (such as aromatic amines and polycyclic aromatic hydrocarbons) in tobacco, which can directly stimulate and damage bladder epithelial cells[108]. Male smokers have been shown to have higher levels of N-nitroso compounds in their urine, which directly induce DNA damage in bladder epithelial cells[109]. It is important to note that men have a higher susceptibility to smoking-related genes, resulting in a weaker ability to detoxify aromatic amines in tobacco, which may further increase the risk of BCa[108].

Occupational exposure is the second leading risk factor for BCa, and men are more likely to be exposed to carcinogens due to traditional occupations such as industry and manufacturing, but women may also be at increased risk in certain industries such as textiles[110,111]. Men are at higher risk of exposure to aromatic amines such as β-naphthylamine and benzidine in industries such as dyes, rubber, and petrochemicals, which can be metabolized by N-hydroxylation to form DNA-reactive intermediates that directly lead to bladder epithelial cell mutations[110].

## 4.2 Drinking and eating habits

The association between alcohol intake and the risk of urinary tract cancers showed significant sex heterogeneity. Cohort studies have shown an inverted U-shaped association between alcohol consumption and BCa risk in men: light to moderate alcohol consumption (151 to 300 g/week of pure ethanol) significantly increases the risk of developing the disease by 67%, but the risk of excessive alcohol consumption (>450 g/week) decreases to below baseline[112]. Notably, there was no significant association between alcohol intake and BCa risk in women[113].

In addition, there is a sex bias in the effect of alcohol type on different tumors. Long-term alcohol consumption (40% ≥ethanol) was positively associated with melanoma and basal cell carcinoma risk in men, while only a slightly increased risk of basal cell carcinoma was observed in women[114]. Mechanistic studies have shown that acetaldehyde dehydrogenase (ALDH2) gene polymorphisms (such as rs671) are more likely to lead to acetaldehyde accumulation in males, which promotes mutations through DNA adduct formation. In women, the upregulation of ALDH2 activity by estrogen may partially counteract the genotoxicity of alcohol metabolites[115]. These findings suggest that sex differences in alcohol-related carcinogenic risk are not only due to behavioral factors, but also closely related to the interaction of metabolic pathways and genetic predisposition.

The protective or promoting effect of dietary components on urinary tract tumors is also sex-specific. In a cohort study, total vegetable and non-starchy vegetable intake were significantly inversely associated with BCa risk in women, but no similar association was found in men[116]. A high intake of red/processed meat in men significantly increases the risk of BCa, while a weaker association is associated in women, possibly with androgen-promoting inflammatory effects[117].In addition, an increase of 100 g of fruit per day in women reduced the risk of non-muscle-invasive bladder cancer (NMIBC) by 28%, while there was no significant protective association in men[118]. These findings suggest that sex differences not only exist in carcinogen exposure levels, but are also deeply rooted in the interaction between nutrient metabolism and molecular regulatory networks, which provides a key basis for the development of sex-stratified dietary intervention strategies.

# 5. Clinical Translation and Therapeutic Strategies

## 5.1 Sex Differences in Response to Immunotherapy

ICIs exhibit differential effects for treatment in different sexs[119,120], and this difference may be associated with a stronger T-cell immune response in men, while the immune system in women is more inclined to a regulatory immune response[121].PD-1 is predominantly expressed on the surface of activated T cells, while PD-L1 is predominantly expressed on tumor cells and antigen-presenting cells[122]. Activation of the PD-1/PD-L1 pathway can inhibit the activity of T cells, thereby helping tumor cells evade the immune system[123].PD-1 inhibitors have been reported to be more effective in male patients with non-small cell lung cancer (NSCLC), especially in populations with high PD-L1 expression, while other immunological combination strategies may need to be explored in female patients[124]. Several pivotal clinical trials have revealed significant sex-based differences in the efficacy of ICIs among patients with advanced urothelial carcinoma[139]. In the phase III KEYNOTE-045 trial, pembrolizumab demonstrated a clear survival advantage in male patients (HR = 0.73, 95% CI 0.59–0.90), whereas no significant survival benefit was observed in female patients (HR = 0.92, 95% CI 0.63–1.33)[140]. A notable sex imbalance was also present in the first-line cohort of the IMvigor210 trial, where male patients constituted 81% of the enrolled population[141] . This enrollment distribution not only reflects the epidemiological characteristics of bladder cancer but also provides a relevant context for assessing potential sex-associated disparities in treatment outcomes[141]. These findings are consistent with a large meta-analysis that confirmed significantly better survival in male patients treated with PD-1/PD-L1 inhibitors compared to females (P = 0.0019), with pooled hazard ratios of 0.72 (95% CI 0.65–0.79) for men and 0.86 (95% CI 0.79–0.93) for women[139]. The concordance across studies underscores sex as a crucial biological variable influencing response to immunotherapy in bladder cancer. The underlying mechanism may be attributed to the significant role of the androgen–androgen receptor signaling axis in shaping a sex-dimorphic tumor immune microenvironment. This pathway promotes the differentiation of CD8⁺ T cells toward an exhausted phenotype in males and upregulates the expression of immune checkpoint molecules such as PD-1, collectively fostering a more immunosuppressive microenvironment[5]. In summary, accumulating evidence strongly indicates that patient sex is a key biological variable affecting responses to immunotherapy. Future clinical practice and trial designs should incorporate sex as an important factor in patient stratification and therapeutic decision-making.

## 5.2 Targeting Hormonal Pathways in Urologic Cancers

Sex hormone signaling pathways are known to play a key role in sex differences in a variety of cancers, and the sex of patients needs to be fully considered in these treatments that target the signaling pathways. Male patients with BCa benefit more from AR signaling inhibition, while female patients are more concerned about estrogen receptor-related pathways[125], Targeting ARs demonstrates promising therapeutic potential[143]. Preclinical and early-phase clinical studies indicate that AR antagonists may inhibit tumor progression directly while also reshaping the immune microenvironment. Their combination with immune checkpoint inhibitors could enhance T-cell activity via ADT, thereby producing synergistic antitumor effects [143,144]. In female patients and specific subtypes of prostate cancer unique to males, modulation of ER signaling has emerged as another critical focus[145,146]. However, the biological role of ERβ in bladder cancer remains controversial; the complexity of its mechanistic actions significantly influences the strategic direction of targeted therapy development. Existing data suggest that ERβ may facilitate proliferation and invasion of bladder cancer cells by regulating the miR-92a/DAB2IP signaling axis, implying a potential oncogenic role[147]. Nevertheless, the proposed “tumor suppressor” or immunomodulatory properties observed in other malignancies remain insufficiently substantiated in bladder cancer models [148].

Amidst these uncertainties, current therapeutic strategies targeting ERβ exhibit marked asymmetry. Present research primarily emphasizes ERβ inhibition, utilizing SERMs to overcome cisplatin resistance in bladder cancer [149]. Conversely, therapeutic approaches aimed at harnessing ERβ’s putative tumor-suppressive or immunomodulatory functions through agonism remain largely exploratory, hindered by a lack of robust supporting evidence, and their clinical viability remains undefined. A central unresolved question is whether ERβ acts as a promoter or suppressor of tumorigenesis in bladder cancer. Future investigations must aim to elucidate its precise functional role, thereby laying a theoretical foundation and guiding translational efforts toward precision-targeted therapies.

## 5.3 Gender-specific therapeutic targets LOY and KDM6A

The loss of the Y chromosome (LOY) is not only a common occurrence in male cancers but also a critical driver of immunosuppressive tumor microenvironment formation. This underscores the potential of targeting LOY-related pathways as a novel, male-centric therapeutic approach[150]. Such strategies may include restoring the function of Y-linked tumor suppressor genes or blocking LOY-induced immunosuppression[151]. A compelling application of this principle is the combination of immunotherapy with gender-based patient stratification; specifically, prioritizing PD-1 or PD-L1 inhibitors in male patients with documented LOY holds promise for improving treatment outcomes by mitigating the distinct immunological and genomic vulnerabilities characteristic of male cancers[150].

Compounding this, gender disparities in cancer susceptibility are further exemplified by KDM6A, an X-linked gene whose expression profile is markedly influenced by sex. While females carry two KDM6A alleles, X-chromosome inactivation limits its expression to approximately half of their cells. In contrast, males, with only a single functional KDM6A copy, face a heightened risk of functional compromise[152]. This genetic asymmetry likely underpins the elevated incidence of specific cancers, including bladder and colorectal cancer, in men[152,153]. At a mechanistic level, KDM6A loss perturbs the balance between activating COMPASS and repressive polycomb complexes, thereby remodeling chromatin and promoting the expression of oncogenic drivers such as IGF1 and ELN[153]. Together, these findings lay a solid theoretical groundwork for advancing male-specific cancer therapies

## 5.4 Gaps in the development of sex-specific biomarkers

Current research has yet to establish a systematic and in-depth understanding of the biological mechanisms underlying sex-based differences in disease, a knowledge gap that is particularly evident in non-reproductive cancers, neurodegenerative disorders, and autoimmune conditions[154]. Compounding this issue, the frequent oversight of sex as a biological variable in study design, coupled with insufficient application of sophisticated analytical methods, has resulted in a persistent shortage of rigorously validated and clinically actionable sex-specific biomarkers[155].This critical deficit ultimately impedes the development and implementation of sex-informed strategies for precision prevention, diagnosis, and treatment.

# 6. Conclusions and prospects

Urinary cancers demonstrate significant sex-based disparities, with males generally exhibiting higher morbidity and mortality rates. These differences arise from a complex interplay between intrinsic biological mechanisms and extrinsic environmental factors. Key contributing mechanisms include the divergent roles of sex hormones—where androgens frequently promote tumor progression, whereas estrogens may confer protective effects—as well as genetic and epigenetic influences, such as biallelic expression of X‑linked tumor suppressors in females and LOY in males. Furthermore, sex‑specific modulation of the immune microenvironment and microbiota profoundly influences tumor development and therapeutic response. Clinically, these biological distinctions directly shape treatment outcomes, as evidenced by sex‑stratified efficacy data from pivotal immunotherapy trials. Therefore, incorporating sex as a fundamental biological variable into research frameworks, therapeutic strategies, and public health initiatives is essential for advancing precision oncology.

# To further the development of sex‑informed precision oncology, coordinated efforts across multiple domains will be required. Future research should prioritize elucidating the complex interactions between sex hormones and sex chromosomes, and rigorously validate emerging sex‑specific therapeutic targets, such as the androgen receptor pathway and Y‑chromosome loss mechanisms in males. Translational efforts must focus on developing sex‑stratified immunotherapeutic approaches and clinically applicable biomarkers, while ensuring sex‑balanced enrollment and pre‑specified sex‑based analyses in clinical trials. At a systemic level, establishing dedicated sex‑specific biobanks and integrating multi‑omics datasets will provide crucial infrastructure. Ultimately, only by embedding the biology of sex differences throughout the continuum of cancer research and clinical care can we achieve truly personalized, equitable, and effective oncology practice.List of abbreviations

| BCa | Bladder Cancer |
| --- | --- |
| AR | Androgen Receptor |
| DHT | Dihydrotestosterone |
| EPPK1 | Epiplakin1 |
| JUP | Junction Plakoglobin |
| MAPK | Mitogen-Activated Protein Kinase |
| TGF-β | Transforming Growth Factor Beta |
| TβRI | TGF-β Receptor I |
| IGF1 | Insulin-like Growth Factor 1 |
| PARP | Poly ADP-ribose Polymerase |
| BER | Base Excision Repair |
| NHEJ | Non-Homologous End Joining |
| ERα | Estrogen Receptor Alpha |
| ERβ | Estrogen Receptor Beta |
| SERM | Selective Estrogen Receptor Modulator |
| MIBC | Muscle-Invasive Bladder Cancer |
| NMIBC | Non-Muscle-Invasive Bladder Cancer |
| PD-1 | Programmed Cell Death Protein 1 |
| PD-L1 | Programmed Death-Ligand 1 |
| ADT | Androgen Deprivation Therapy |
| GPER1 | G Protein-Coupled Estrogen Receptor 1 |
| VEGF | Vascular Endothelial Growth Factor |
| Th1 | T Helper 1 Cell |
| Th2 | T Helper 2 Cell |
| Treg | Regulatory T Cell |
| XCI | X Chromosome Inactivation |
| EXITS | Escape from X-Inactivation Tumor Suppressors |
| LOY | Loss of Y Chromosome |
| mLOY | Mosaic Loss of Y Chromosome |
| CRC | Colon Cancer |
| XIST | X-Inactive Specific Transcript |
| HCC | Hepatocellular Carcinoma |
| lncRNA | Long Non-Coding RNA |
| circRNA | Circular RNA |
| LUTS | Lower Urinary Tract Symptoms |
| ICI | Immune Checkpoint Inhibitor |
| NSCLC | Non-Small Cell Lung Cancer |
| ALDH2 | Aldehyde Dehydrogenase 2 |

# Declarations:

**Ethics approval**

Not applicable

**Consent to publicate**

Not applicable

**Consent to publish**

All authors have read and agree to publish

# Availability of data and materials

Not applicable

# Competing interests

The authors declare that they have no competing interests

# Funding

This study was supported by the Jiangxi Provincial Key R&D Program (No. 20212BBG71013), Ganzhou Science and Technology Innovation Talent Project (No. 2022CXRC9621), Jiangxi Department of Education Science and Technology Project (No. GJJ190819), Ganzhou Science and Technology Guidance Program (No. GZ2023ZSF097)，and the Jiangxi Graduate Student Innovation Fund (No. YC2025-S234).

# Authors' contributions

PF and DW contributed equally to this work as co-first authors. They were responsible for the core conception of the study, conducted extensive literature research, drafted the manuscript, and coordinated the writing process. GS,YW,SY, YX,PX and ZL contributed to literature collection, data curation. JZ provided critical feedback on the manuscript structure and clinical relevance. XZ, as the corresponding author, conceived the study, supervised the entire project, provided guidance, reviewed the manuscript, and finalized the content. All authors read and approved the final manuscript.

# References

1. Schafer EJ, Jemal A, Wiese D, et al. Disparities and Trends in Genitourinary Cancer Incidence and Mortality in the USA. *Eur Urol*. 2023;84(1):117-126. doi:10.1016/j.eururo.2022.11.023

2. Siegel RL, Kratzer TB, Giaquinto AN, Sung H, Jemal A. Cancer statistics, 2025. *Ca*. 2025;75(1):10. doi:10.3322/caac.21871

3. Conforti F, Pala L, Di Mitri D, et al. Sex hormones, the anticancer immune response, and therapeutic opportunities. *Cancer Cell*. 2025;43(3):343-360. doi:10.1016/j.ccell.2025.02.013

4. Wang L, Shilatifard A. UTX Mutations in Human Cancer. *Cancer Cell*. 2019;35(2):168-176. doi:10.1016/j.ccell.2019.01.001

5. Kwon H, Schafer JM, Song NJ, et al. Androgen Conspires with the CD8+ T Cell Exhaustion Program and Contributes to Sex Bias in Cancer. *Sci Immunol*. 2022;7(73):eabq2630. doi:10.1126/sciimmunol.abq2630

6. Wang J, Li D, Wu R, Feng D. Cutting‐Edge Advancements in the Antibiotics‐Gut Microbiota‐Urinary Tumour Axis. *Cell Prolif*. 2025;58(5):e70023. doi:10.1111/cpr.70023

7. Prall OW, Sarcevic B, Musgrove EA, Watts CK, Sutherland RL. Estrogen-induced activation of Cdk4 and Cdk2 during G1-S phase progression is accompanied by increased cyclin D1 expression and decreased cyclin-dependent kinase inhibitor association with cyclin E-Cdk2. *J Biol Chem*. 1997;272(16):10882-10894. doi:10.1074/jbc.272.16.10882

8. Rim EY, Clevers H, Nusse R. The Wnt Pathway: From Signaling Mechanisms to Synthetic Modulators. *Annu Rev Biochem*. 2022;91:571-598. doi:10.1146/annurev-biochem-040320-103615

9. Yedidia-Aryeh L, Goldberg M. The Interplay between the Cellular Response to DNA Double-Strand Breaks and Estrogen. *Cells*. 2022;11(19):3097. doi:10.3390/cells11193097

10. Abdel-Hafiz HA, Schafer JM, Chen X, et al. Y chromosome loss in cancer drives growth by evasion of adaptive immunity. *Nature*. 2023;619(7970):624-631. doi:10.1038/s41586-023-06234-x

11. Ece G, Aktaş A, Caner A, et al. The Urogenital System Microbiota: Is It a New Gamechanger in Urogenital Cancers? *Microorganisms*. 2025;13(2):315. doi:10.3390/microorganisms13020315

12. Kouzmenko AP, Takeyama K ichi, Ito S, et al. Wnt/β-Catenin and Estrogen Signaling Converge in Vivo*. *Journal of Biological Chemistry*. 2004;279(39):40255-40258. doi:10.1074/jbc.C400331200

13. Somjen D, Weisman Y, Kohen F, et al. 25-hydroxyvitamin D3-1alpha-hydroxylase is expressed in human vascular smooth muscle cells and is upregulated by parathyroid hormone and estrogenic compounds. *Circulation*. 2005;111(13):1666-1671. doi:10.1161/01.CIR.0000160353.27927.70

14. Corachán A, Ferrero H, Aguilar A, et al. Inhibition of tumor cell proliferation in human uterine leiomyomas by vitamin D via Wnt/β-catenin pathway. *Fertility and Sterility*. 2019;111(2):397-407. doi:10.1016/j.fertnstert.2018.10.008

15. Krishnan AV, Swami S, Feldman D. Vitamin D and breast cancer: Inhibition of estrogen synthesis and signaling. *The Journal of Steroid Biochemistry and Molecular Biology*. 2010;121(1):343-348. doi:10.1016/j.jsbmb.2010.02.009

16. Chen CR, Kang Y, Siegel PM, Massagué J. E2F4/5 and p107 as Smad Cofactors Linking the TGFβ Receptor to c-myc Repression. *Cell*. 2002;110(1):19-32. doi:10.1016/S0092-8674(02)00801-2

17. Wang Y, Tong X, Xiao Y, et al. Regulating Integrin β1 to Restore Gonadotropin-Releasing Hormone–Tanycyte Unit Function in Polycystic Ovary Syndrome-Related Hypothalamic Dysregulation. *Research (Wash D C)*. 8:0619. doi:10.34133/research.0619

18. Seoane J, Gomis RR. TGF-β Family Signaling in Tumor Suppression and Cancer Progression. *Cold Spring Harb Perspect Biol*. 2017;9(12):a022277. doi:10.1101/cshperspect.a022277

19. Alsamraae M, Costanzo-Garvey D, Teply BA, et al. Androgen receptor inhibition suppresses anti-tumor neutrophil response against bone metastatic prostate cancer via regulation of TβRI expression. *Cancer Lett*. 2023;579:216468. doi:10.1016/j.canlet.2023.216468

20. Yang L, Huang W, Bai X, et al. Androgen dihydrotestosterone promotes bladder cancer cell proliferation and invasion via EPPK1-mediated MAPK/JUP signalling. *Cell Death Dis*. 2023;14(6):363. doi:10.1038/s41419-023-05882-1

21. Hu Y dou, Wu K, Liu Y jie, et al. LY6/PLAUR domain containing 3 (LYPD3) maintains melanoma cell stemness and mediates an immunosuppressive microenvironment. *Biol Direct*. 2023;18:72. doi:10.1186/s13062-023-00424-3

22. Chen J, Huang CP, Quan C, et al. The androgen receptor in bladder cancer. *Nat Rev Urol*. 2023;20(9):560-574. doi:10.1038/s41585-023-00761-y

23. Chi R, Liu Y, Wang P, et al. Estrogen-induced circFAM171A1 regulates sheep myoblast proliferation through the oar-miR-485-5p/MAPK15/MAPK pathway. *Cell Mol Life Sci*. 2025;82(1):123. doi:10.1007/s00018-025-05639-3

24. Palominos C, Fuentes-Retamal S, Salazar JP, et al. Mitochondrial bioenergetics as a cell fate rheostat for responsive to Bcl-2 drugs: New cues for cancer chemotherapy. *Cancer Letters*. 2024;594:216965. doi:10.1016/j.canlet.2024.216965

25. Wu J, Li J, Liu Y, et al. Tannic acid repair of zearalenone-induced damage by regulating the death receptor and mitochondrial apoptosis signaling pathway in mice. *Environmental Pollution*. 2021;287:117557. doi:10.1016/j.envpol.2021.117557

26. Adzavon YM, Culig Z, Sun Z. Interactions between androgen and IGF1 axes in prostate tumorigenesis. *Nat Rev Urol*. Published online October 7, 2024. doi:10.1038/s41585-024-00942-3

27. Westaby D, Fenor de La Maza M de LD, Paschalis A, et al. A New Old Target: Androgen Receptor Signaling and Advanced Prostate Cancer. *Annu Rev Pharmacol Toxicol*. 2022;62:131-153. doi:10.1146/annurev-pharmtox-052220-015912

28. Gilbert S, Péant B, Malaquin N, et al. Targeting IKKε in Androgen-Independent Prostate Cancer Causes Phenotypic Senescence and Genomic Instability. *Mol Cancer Ther*. 2022;21(3):407-418. doi:10.1158/1535-7163.MCT-21-0519

29. Rajan A, Varghese GR, Yadev I, et al. Modulation of BRCA1 mediated DNA damage repair by deregulated ER-α signaling in breast cancers. *Am J Cancer Res*. 2022;12(1):17-47.

30. Rangsrikitphoti P, Marquez-Garban DC, Pietras RJ, McGowan E, Boonyaratanakornkit V. Sex steroid hormones and DNA repair regulation: Implications on cancer treatment responses. *The Journal of Steroid Biochemistry and Molecular Biology*. 2023;227:106230. doi:10.1016/j.jsbmb.2022.106230

31. Wang J, Li C, Han J, et al. Reassessing the roles of oxidative DNA base lesion 8-oxoGua and repair enzyme OGG1 in tumorigenesis. *J Biomed Sci*. 2025;32:1. doi:10.1186/s12929-024-01093-8

32. Bhardwaj P, Iyengar NM, Zahid H, et al. Obesity promotes breast epithelium DNA damage in women carrying a germline mutation in BRCA1 or BRCA2. *Sci Transl Med*. 2023;15(684):eade1857. doi:10.1126/scitranslmed.ade1857

33. Wu W, Zhu J, Nihira NT, et al. Ribosomal S6 kinase (RSK) plays a critical role in DNA damage response via the phosphorylation of histone lysine demethylase KDM4B. *Breast Cancer Res*. 2024;26:146. doi:10.1186/s13058-024-01901-x

34. Ueda T, Kanai A, Komuro A, et al. KDM4B promotes acute myeloid leukemia associated with AML1‐ETO by regulating chromatin accessibility. *FASEB Bioadv*. 2021;3(12):1020-1033. doi:10.1096/fba.2021-00030

35. Shafi AA, McNair CM, McCann JJ, et al. The circadian cryptochrome, CRY1, is a pro-tumorigenic factor that rhythmically modulates DNA repair. *Nat Commun*. 2021;12:401. doi:10.1038/s41467-020-20513-5

36. Hasterok S, Scott TG, Roller DG, et al. The androgen receptor does not directly regulate the transcription of DNA damage response genes. *Mol Cancer Res*. 2023;21(12):1329-1341. doi:10.1158/1541-7786.MCR-23-0358

37. Ganguly S, Lone Z, Muskara A, et al. Intratumoral androgen biosynthesis associated with 3β-hydroxysteroid dehydrogenase 1 promotes resistance to radiotherapy in prostate cancer. *J Clin Invest*. 133(22):e165718. doi:10.1172/JCI165718

38. Talazoparib plus enzalutamide in men with first-line metastatic castration-resistant prostate cancer (TALAPRO-2): a randomised, placebo-controlled, phase 3 trial. *The Lancet*. 2023;402(10398):291-303. doi:10.1016/S0140-6736(23)01055-3

39. Teng J, Wang ZY, Jarrard DF, Bjorling DE. Roles of estrogen receptor α and β in modulating urothelial cell proliferation. *Endocr Relat Cancer*. 2008;15(1):351-364. doi:10.1677/erc.1.01255

40. Ide H, Inoue S, Miyamoto H. Histopathological and prognostic significance of the expression of sex hormone receptors in bladder cancer: A meta-analysis of immunohistochemical studies. *PLoS One*. 2017;12(3):e0174746. doi:10.1371/journal.pone.0174746

41. Di Zazzo E, Galasso G, Giovannelli P, et al. Estrogen Receptors in Epithelial-Mesenchymal Transition of Prostate Cancer. *Cancers (Basel)*. 2019;11(10):1418. doi:10.3390/cancers11101418

42. Belluti S, Imbriano C, Casarini L. Nuclear Estrogen Receptors in Prostate Cancer: From Genes to Function. *Cancers (Basel)*. 2023;15(18):4653. doi:10.3390/cancers15184653

43. Li J, Liu Q, Jiang C. Signal Crosstalk and the Role of Estrogen Receptor beta (ERβ) in Prostate Cancer. *Med Sci Monit*. 2022;28:e935599-1-e935599-7. doi:10.12659/MSM.935599

44. Ramírez-de-Arellano A, Pereira-Suárez AL, Rico-Fuentes C, López-Pulido EI, Villegas-Pineda JC, Sierra-Diaz E. Distribution and Effects of Estrogen Receptors in Prostate Cancer: Associated Molecular Mechanisms. *Front Endocrinol (Lausanne)*. 2022;12:811578. doi:10.3389/fendo.2021.811578

45. Yan S, Wang J, Chen H, Zhang D, Imam M. Divergent features of ERβ isoforms in triple negative breast cancer: progress and implications for further research. *Front Cell Dev Biol*. 2023;11:1240386. doi:10.3389/fcell.2023.1240386

46. Jefferi NES, Shamhari A ‘Afifah, Noor Azhar NKZ, et al. The Role of ERα and ERβ in Castration-Resistant Prostate Cancer and Current Therapeutic Approaches. *Biomedicines*. 2023;11(3):826. doi:10.3390/biomedicines11030826

47. Liu Y, Ma H, Yao J. ER&alpha;, A Key Target for Cancer Therapy: A Review. *OTT*. 2020;13:2183-2191. doi:10.2147/OTT.S236532

48. Sikic D, Taubert H, Wirtz RM, et al. High Androgen Receptor mRNA Expression Is Associated with Improved Outcome in Patients with High-Risk Non-Muscle-Invasive Bladder Cancer. *Life (Basel)*. 2021;11(7):642. doi:10.3390/life11070642

49. Sottnik JL, Vanderlinden L, Joshi M, et al. Androgen Receptor Regulates CD44 Expression in Bladder Cancer. *Cancer Res*. 2021;81(11):2833-2846. doi:10.1158/0008-5472.CAN-20-3095

50. De Ieso ML, Aldoghachi AF, Tilley WD, Dwyer AR. Are androgen receptor agonists a treatment option in bladder cancer? *The Journal of Steroid Biochemistry and Molecular Biology*. 2025;245:106623. doi:10.1016/j.jsbmb.2024.106623

51. Sun A, Luo Y, Xiao W, et al. Androgen Receptor Transcriptionally Inhibits Programmed Death Ligand-1 Expression and Influences Immune Escape in Bladder Cancer. *Laboratory Investigation*. 2023;103(7). doi:10.1016/j.labinv.2023.100148

52. Lim CL, Or YZ, Ong Z, et al. Estrogen exacerbates mammary involution through neutrophil-dependent and -independent mechanism. *eLife*. 9:e57274. doi:10.7554/eLife.57274

53. Pettersson C, Wu R, Demirel I. Estrogen-stimulated uropathogenic E. coli mediate enhanced neutrophil responses. *Sci Rep*. 2024;14:23030. doi:10.1038/s41598-024-74863-x

54. Lim CL, Lin VCL. Estrogen markedly reduces circulating low-density neutrophils and enhances pro-tumoral gene expression in neutrophil of tumour-bearing mice. *BMC Cancer*. 2021;21:1017. doi:10.1186/s12885-021-08751-2

55. Estrogen receptors in urogenital schistosomiasis and bladder cancer: Estrogen receptor alpha-mediated cell proliferation. *Urologic Oncology: Seminars and Original Investigations*. 2020;38(9):738.e23-738.e35. doi:10.1016/j.urolonc.2020.04.022

56. Yajuk O, Baron M, Toker S, Zelter T, Fainsod-Levi T, Granot Z. The PD-L1/PD-1 Axis Blocks Neutrophil Cytotoxicity in Cancer. *Cells*. 2021;10(6):1510. doi:10.3390/cells10061510

57. Zhang X, Cheng L, Gao C, et al. Androgen Signaling Contributes to Sex Differences in Cancer by Inhibiting NF-κB Activation in T Cells and Suppressing Antitumor Immunity. *Cancer Res*. 2023;83(6):906-921. doi:10.1158/0008-5472.CAN-22-2405

58. Pala L, De Pas T, Conforti F. Boosting anticancer immunotherapy through androgen receptor blockade. *Cancer Cell*. 2022;40(5):455-457. doi:10.1016/j.ccell.2022.04.007

59. Chen S, Saeed AFUH, Liu Q, et al. Macrophages in immunoregulation and therapeutics. *Signal Transduct Target Ther*. 2023;8:207. doi:10.1038/s41392-023-01452-1

60. Locati M, Curtale G, Mantovani A. Diversity, Mechanisms and Significance of Macrophage Plasticity. *Annu Rev Pathol*. 2020;15:123-147. doi:10.1146/annurev-pathmechdis-012418-012718

61. Artham S, Chang CY, McDonnell DP. Eosinophilia in cancer and its regulation by sex hormones. *Trends Endocrinol Metab*. 2023;34(1):5-20. doi:10.1016/j.tem.2022.11.002

62. Yang Y, Wang Y, Zou H, et al. GPER1 signaling restricts macrophage proliferation and accumulation in human hepatocellular carcinoma. *Front Immunol*. 2024;15:1481972. doi:10.3389/fimmu.2024.1481972

63. Adachi A, Honda T, Egawa G, et al. Estradiol suppresses psoriatic inflammation in mice by regulating neutrophil and macrophage functions. *J Allergy Clin Immunol*. 2022;150(4):909-919.e8. doi:10.1016/j.jaci.2022.03.028

64. Barcena ML, Christiansen-Mensch C, Aslam M, Haritonow N, Ladilov Y, Regitz-Zagrosek V. Upregulation of Mitochondrial Sirt3 and Alleviation of the Inflammatory Phenotype in Macrophages by Estrogen. *Cells*. 2024;13(17):1420. doi:10.3390/cells13171420

65. Bakhshi P, Ho JQ, Zanganeh S. Sex-specific outcomes in cancer therapy: the central role of hormones. *Frontiers in Medical Technology*. 2024;6:1320690. doi:10.3389/fmedt.2024.1320690

66. Hreha TN, Collins CA, Daugherty AL, Griffith JM, Hruska KA, Hunstad DA. Androgen-Influenced Polarization of Activin A-Producing Macrophages Accompanies Post-pyelonephritic Renal Scarring. *Front Immunol*. 2020;11:1641. doi:10.3389/fimmu.2020.01641

67. Rehman A, Pacher P, Haskó G. Role of Macrophages in the Endocrine System. *Trends in Endocrinology & Metabolism*. 2021;32(4):238-256. doi:10.1016/j.tem.2020.12.001

68. El-Kenawi A, Dominguez-Viqueira W, Liu M, et al. Macrophage-derived cholesterol contributes to therapeutic resistance in prostate cancer. *Cancer Res*. 2021;81(21):5477-5490. doi:10.1158/0008-5472.CAN-20-4028

69. Cioni B, Zaalberg A, van Beijnum JR, et al. Androgen receptor signalling in macrophages promotes TREM-1-mediated prostate cancer cell line migration and invasion. *Nat Commun*. 2020;11:4498. doi:10.1038/s41467-020-18313-y

70. Hreha TN, Collins CA, Cole EB, Jin RJ, Hunstad DA. Androgen exposure impairs neutrophil maturation and function within the infected kidney. *mBio*. 15(2):e03170-23. doi:10.1128/mbio.03170-23

71. Artham S, Chang CY, McDonnell DP. Eosinophilia in cancer and its regulation by sex hormones. *Trends Endocrinol Metab*. 2023;34(1):5-20. doi:10.1016/j.tem.2022.11.002

72. Sun A, Luo Y, Xiao W, et al. Androgen Receptor Transcriptionally Inhibits Programmed Death Ligand-1 Expression and Influences Immune Escape in Bladder Cancer. *Laboratory Investigation*. 2023;103(7). doi:10.1016/j.labinv.2023.100148

73. Deltourbe L, Lacerda Mariano L, Hreha TN, Hunstad DA, Ingersoll MA. The impact of biological sex on diseases of the urinary tract. *Mucosal Immunol*. 2022;15(5):857-866. doi:10.1038/s41385-022-00549-0

74. Yang C, Jin J, Yang Y, et al. Androgen receptor-mediated CD8+ T cell stemness programs drive sex differences in antitumor immunity. *Immunity*. 2022;55(7):1268-1283.e9. doi:10.1016/j.immuni.2022.05.012

75. Androgen Receptor Signaling Reduces Male Antitumor CD8+ T-cell Activity. *Cancer Discov*. 2022;12(8):1836. doi:10.1158/2159-8290.CD-RW2022-117

76. Zhang X, Cheng L, Gao C, et al. Androgen Signaling Contributes to Sex Differences in Cancer by Inhibiting NF-κB Activation in T Cells and Suppressing Antitumor Immunity. *Cancer Research*. 2023;83(6):906-921. doi:10.1158/0008-5472.CAN-22-2405

77. Pinto JA, Araujo JM, Gómez HL. Sex, immunity, and cancer. *Biochimica et Biophysica Acta (BBA) - Reviews on Cancer*. 2022;1877(1):188647. doi:10.1016/j.bbcan.2021.188647

78. Caramia F, Speed TP, Shen H, Haupt Y, Haupt S. Establishing the Link between X-Chromosome Aberrations and TP53 Status, with Breast Cancer Patient Outcomes. *Cells*. 2023;12(18):2245. doi:10.3390/cells12182245

79. Wang D, Tang L, Wu Y, et al. Abnormal X chromosome inactivation and tumor development. *Cell Mol Life Sci*. 2020;77(15):2949-2958. doi:10.1007/s00018-020-03469-z

80. Dunford A, Weinstock DM, Savova V, et al. Tumor suppressor genes that escape from X-inactivation contribute to cancer sex bias. *Nat Genet*. 2017;49(1):10-16. doi:10.1038/ng.3726

81. Cáceres A, Jene A, Esko T, Pérez-Jurado LA, González JR. Extreme Downregulation of Chromosome Y and Cancer Risk in Men. *J Natl Cancer Inst*. 2020;112(9):913-920. doi:10.1093/jnci/djz232

82. Li X, Zhang Y, Zheng L, Liu M, Chen CD, Jiang H. UTX is an escape from X-inactivation tumor-suppressor in B cell lymphoma. *Nat Commun*. 2018;9:2720. doi:10.1038/s41467-018-05084-w

83. Bruhn-Olszewska B, Markljung E, Rychlicka-Buniowska E, Sarkisyan D, Filipowicz N, Dumanski JP. The effects of loss of Y chromosome on male health. *Nat Rev Genet*. 2025;26(5):320-335. doi:10.1038/s41576-024-00805-y

84. Loftfield E, Zhou W, Yeager M, Chanock SJ, Freedman ND, Machiela MJ. Mosaic Y loss is moderately associated with solid tumor risk. *Cancer Res*. 2019;79(3):461-466. doi:10.1158/0008-5472.CAN-18-2566

85. Qin N, Li N, Wang C, et al. Association of Mosaic Loss of Chromosome Y with Lung Cancer Risk and Prognosis in a Chinese Population. *Journal of Thoracic Oncology*. 2019;14(1):37-44. doi:10.1016/j.jtho.2018.09.013

86. Wright DJ, Day FR, Kerrison ND, et al. Genetic variants associated with mosaic Y chromosome loss highlight cell cycle genes and overlap with cancer susceptibility. *Nat Genet*. 2017;49(5):674-679. doi:10.1038/ng.3821

87. Köferle A, Schlattl A, Hörmann A, et al. Interrogation of cancer gene dependencies reveals paralog interactions of autosome and sex chromosome-encoded genes. *Cell Reports*. 2022;39(2). doi:10.1016/j.celrep.2022.110636

88. Maan AA, Eales J, Akbarov A, et al. The Y chromosome: a blueprint for men’s health? *Eur J Hum Genet*. 2017;25(11):1181-1188. doi:10.1038/ejhg.2017.128

89. Y Chromosome Loss Drives Bladder Cancer Aggressiveness and Immune Evasion. *Cancer Discov*. 2023;13(8):1761. doi:10.1158/2159-8290.CD-RW2023-102

90. Li J, Lan Z, Liao W, et al. Histone demethylase KDM5D upregulation drives sex differences in colon cancer. *Nature*. 2023;619(7970):632-639. doi:10.1038/s41586-023-06254-7

91. Liu F, Yuan JH, Huang JF, et al. Long noncoding RNA FTX inhibits hepatocellular carcinoma proliferation and metastasis by binding MCM2 and miR-374a. *Oncogene*. 2016;35(41):5422-5434. doi:10.1038/onc.2016.80

92. Liu F, Yuan JH, Huang JF, et al. Long noncoding RNA FTX inhibits hepatocellular carcinoma proliferation and metastasis by binding MCM2 and miR-374a. *Oncogene*. 2016;35(41):5422-5434. doi:10.1038/onc.2016.80

93. Huang C, Azizi P, Vazirzadeh M, et al. Non-coding RNAs/DNMT3B axis in human cancers: from pathogenesis to clinical significance. *J Transl Med*. 2023;21:621. doi:10.1186/s12967-023-04510-y

94. Hua JT, Chen S, He HH. Landscape of Noncoding RNA in Prostate Cancer. *Trends in Genetics*. 2019;35(11):840-851. doi:10.1016/j.tig.2019.08.004

95. Zhu J, Li Q, Wu Z, Xu W, Jiang R. Circular RNA-mediated miRNA sponge & RNA binding protein in biological modulation of breast cancer. *Noncoding RNA Res*. 2024;9(1):262-276. doi:10.1016/j.ncrna.2023.12.005

96. Fan YJ, Ding Z, Zhang Y, et al. Sex-lethal regulates back-splicing and generation of the sex-differentially expressed circular RNAs. *Nucleic Acids Res*. 2023;51(10):5228-5241. doi:10.1093/nar/gkad280

97. Liu S, Lai W, Shi Y, et al. Annotation and cluster analysis of long noncoding RNA linked to male sex and estrogen in cancers. *NPJ Precis Oncol*. 2020;4:5. doi:10.1038/s41698-020-0110-5

98. Li J, Ming Z, Yang L, Wang T, Liu G, Ma Q. Long noncoding RNA XIST: Mechanisms for X chromosome inactivation, roles in sex-biased diseases, and therapeutic opportunities. *Genes Dis*. 2022;9(6):1478-1492. doi:10.1016/j.gendis.2022.04.007

99. Shao T, Xie Y, Shi J, et al. Surveying lncRNA-lncRNA cooperations reveals dominant effect on tumor immunity cross cancers. *Commun Biol*. 2022;5:1324. doi:10.1038/s42003-022-04249-0

100. Li X, Wu Y, Jin Y. Exosomal LncRNAs and CircRNAs in lung cancer: Emerging regulators and potential therapeutic targets. *Noncoding RNA Res*. 2024;9(4):1069-1079. doi:10.1016/j.ncrna.2024.06.010

101. Yu SH, Jung SI. The Potential Role of Urinary Microbiome in Benign Prostate Hyperplasia/Lower Urinary Tract Symptoms. *Diagnostics (Basel)*. 2022;12(8):1862. doi:10.3390/diagnostics12081862

102. Kim MS, Jung SI. The Urinary Tract Microbiome in Male Genitourinary Diseases: Focusing on Benign Prostate Hyperplasia and Lower Urinary Tract Symptoms. *Int Neurourol J*. 2021;25(1):3-11. doi:10.5213/inj.2040174.087

103. Parra-Grande M, Oré-Arce M, Martínez-Priego L, et al. Profiling the Bladder Microbiota in Patients With Bladder Cancer. *Front Microbiol*. 2022;12:718776. doi:10.3389/fmicb.2021.718776

104. Bilski K, Żeber-Lubecka N, Kulecka M, et al. Microbiome Sex-Related Diversity in Non-Muscle-Invasive Urothelial Bladder Cancer. *Curr Issues Mol Biol*. 2024;46(4):3595-3609. doi:10.3390/cimb46040225

105. Heidar NA, Bhat TA, Shabir U, Hussein AA. The Urinary Microbiome and Bladder Cancer. *Life (Basel)*. 2023;13(3):812. doi:10.3390/life13030812

106. Jing N, Wang L, Zhuang H, Ai C, Jiang G, Liu Z. Sex-Biased Immune Responses to Antibiotics during Anti-PD-L1 Treatment in Mice with Colon Cancer. *J Immunol Res*. 2022;2022:9202491. doi:10.1155/2022/9202491

107. Liu F, Han Z, Lu J, Zhong W. Development and validation of a tobacco smoking-related index for predicting overall survival and immunotherapy response in bladder cancer. *Environ Sci Pollut Res Int*. 2023;30(26):68701-68715. doi:10.1007/s11356-023-27132-9

108. Xiong J, Yang L, Deng YQ, et al. The causal association between smoking, alcohol consumption and risk of bladder cancer: A univariable and multivariable Mendelian randomization study. *Int J Cancer*. 2022;151(12):2136-2143. doi:10.1002/ijc.34228

109. Doshi B, Athans SR, Woloszynska A. Biological differences underlying sex and gender disparities in bladder cancer: current synopsis and future directions. *Oncogenesis*. 2023;12(1):44. doi:10.1038/s41389-023-00489-9

110. Shala NK, Stenehjem JS, Babigumira R, et al. Exposure to benzene and other hydrocarbons and risk of bladder cancer among male offshore petroleum workers. *Br J Cancer*. 2023;129(5):838-851. doi:10.1038/s41416-023-02357-0

111. Hosseini B, Zendehdel K, Bouaoun L, et al. Bladder cancer risk in relation to occupations held in a nationwide case-control study in Iran. *Int J Cancer*. 2023;153(4):765-774. doi:10.1002/ijc.34560

112. Masaoka H, Matsuo K, Sawada N, et al. Alcohol consumption and bladder cancer risk with or without the flushing response: The Japan Public Health Center-based Prospective Study. *Int J Cancer*. 2017;141(12):2480-2488. doi:10.1002/ijc.31028

113. Botteri E, Ferrari P, Roswall N, et al. Alcohol consumption and risk of urothelial cell bladder cancer in the European prospective investigation into cancer and nutrition cohort. *Int J Cancer*. 2017;141(10):1963-1970. doi:10.1002/ijc.30894

114. Mahamat‐Saleh Y, Al‐Rahmoun M, Severi G, et al. Baseline and lifetime alcohol consumption and risk of skin cancer in the European Prospective Investigation into Cancer and Nutrition cohort (EPIC). *Int J Cancer*. 2023;152(3):348-362. doi:10.1002/ijc.34253

115. Antwi SO, Eckel-Passow JE, Diehl ND, et al. Alcohol consumption, variability in alcohol dehydrogenase genes and risk of renal cell carcinoma. *Int J Cancer*. 2018;142(4):747-756. doi:10.1002/ijc.31103

116. Yu EYW, Wesselius A, Mehrkanoon S, et al. Vegetable intake and the risk of bladder cancer in the BLadder Cancer Epidemiology and Nutritional Determinants (BLEND) international study. *BMC Med*. 2021;19:56. doi:10.1186/s12916-021-01931-8

117. Dianatinasab M, Wesselius A, Salehi‐Abargouei A, et al. Adherence to a Western dietary pattern and risk of bladder cancer: A pooled analysis of 13 cohort studies of the Bladder Cancer Epidemiology and Nutritional Determinants international study. *Int J Cancer*. 2020;147(12):3394-3403. doi:10.1002/ijc.33173

118. Jochems SHJ, Reulen RC, van Osch FHM, et al. Fruit consumption and the risk of bladder cancer: A pooled analysis by the Bladder Cancer Epidemiology and Nutritional Determinants Study. *Int J Cancer*. 2020;147(8):2091-2100. doi:10.1002/ijc.33008

119. Choi MG, Choi CM, Lee DH, et al. Impact of gender on response to immune checkpoint inhibitors in patients with non-small cell lung cancer undergoing second- or later-line treatment. *Transl Lung Cancer Res*. 2022;11(9):1866-1876. doi:10.21037/tlcr-22-146

120. Klein SL, Flanagan KL. Sex differences in immune responses. *Nat Rev Immunol*. 2016;16(10):626-638. doi:10.1038/nri.2016.90

121. Vavalà T. Immunotherapy outcomes in non-small cell lung cancer according to a gender perspective. *Prog Mol Biol Transl Sci*. 2024;209:241-258. doi:10.1016/bs.pmbts.2024.09.004

122. Singh S, Singh N, Baranwal M, Sharma S, Devi SSK, Kumar S. Understanding immune checkpoints and PD-1/PD-L1-mediated immune resistance towards tumour immunotherapy. *3 Biotech*. 2023;13(12):411. doi:10.1007/s13205-023-03826-2

123. Ashrafizadeh M, Zarrabi A, Hushmandi K, et al. PD-1/PD-L1 axis regulation in cancer therapy: The role of long non-coding RNAs and microRNAs. *Life Sci*. 2020;256:117899. doi:10.1016/j.lfs.2020.117899

124. Conforti F, Pala L, Pagan E, et al. Sex-based differences in response to anti-PD-1 or PD-L1 treatment in patients with non-small-cell lung cancer expressing high PD-L1 levels. A systematic review and meta-analysis of randomized clinical trials. *ESMO Open*. 2021;6(5):100251. doi:10.1016/j.esmoop.2021.100251

125. Tao X, Wang Y, Xiang B, et al. Sex bias in tumor immunity: insights from immune cells. *Theranostics*. 2025;15(11):5045-5072. doi:10.7150/thno.106465

126. Warner M, Huang B, Gustafsson JA. Estrogen Receptor β as a Pharmaceutical Target. *Trends Pharmacol Sci*. 2017;38(1):92-99. doi:10.1016/j.tips.2016.10.006

127. Luo J, Hu Z, Xiao Y, et al. Rational design and optimization of selenophenes with basic side chains as novel potent selective estrogen receptor modulators (SERMs) for breast cancer therapy †Electronic supplementary information (ESI) available: 1H NMR and 13C NMR spectra of final compounds. See DOI: 10.1039/c7md00163k. *Medchemcomm*. 2017;8(7):1485-1497. doi:10.1039/c7md00163k

128. Valentín López JC, Lange CA, Dehm SM. Androgen receptor and estrogen receptor variants in prostate and breast cancers. *J Steroid Biochem Mol Biol*. 2024;241:106522. doi:10.1016/j.jsbmb.2024.106522

129. Belluti S, Imbriano C, Casarini L. Nuclear Estrogen Receptors in Prostate Cancer: From Genes to Function. *Cancers (Basel)*. 2023;15(18):4653. doi:10.3390/cancers15184653


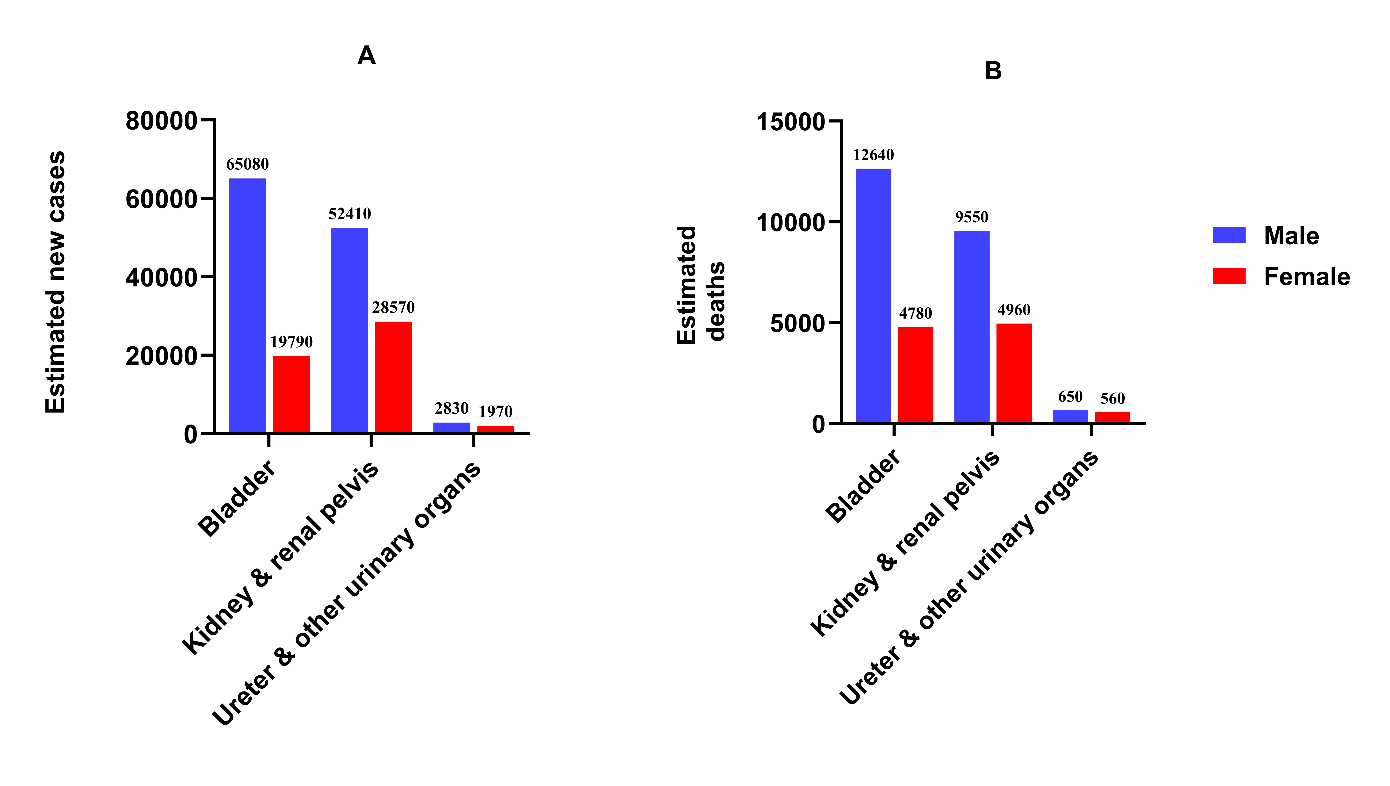


Figure 1 Gender disparities in the estimated number of new cases and deaths for urinary system cancers in the United States, 2025.**(A)**:Estimated new cases.**(B)**:Estimated deaths. Data are presented for bladder cancer, kidney & renal pelvis cancer, and ureter & other urinary organs cancer.

*Data source: Siegel RL, Kratzer TB, Giaquinto AN, Sung H, Jemal A. Cancer statistics, 2025. *Ca*. 2025;75(1):10. doi:10.3322/caac.21871


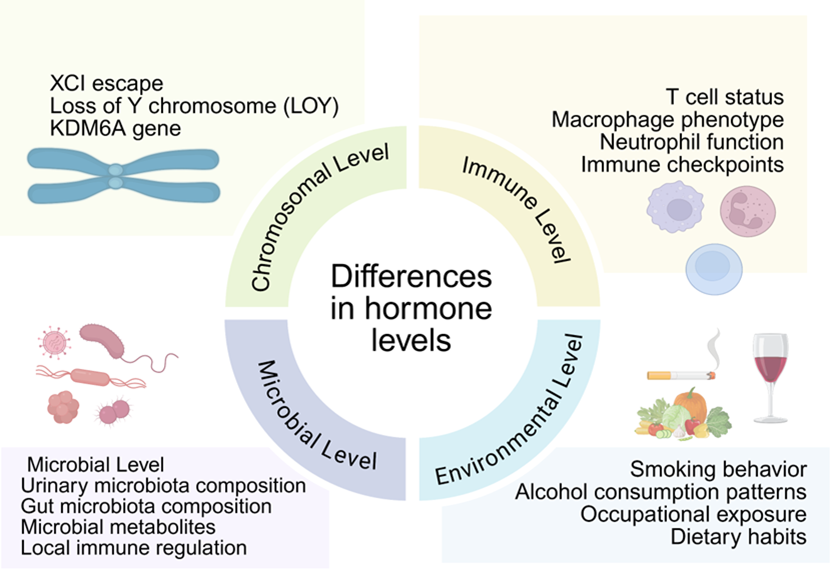


**Fig. 2** Multidimensional mechanisms underlying sex differences in tumor biology.The schematic illustrates the four interconnected dimensions contributing to sex disparities in cancer: chromosomal/epigenetic factors (XCI escape, LOY, KDM6A), immune/hormonal regulation (T cell status, macrophage polarization), microbial/local environment (microbiota composition), and lifestyle/environmental exposures (smoking, diet, occupation). Central to these interactions are intrinsic differences in sex hormone levels.


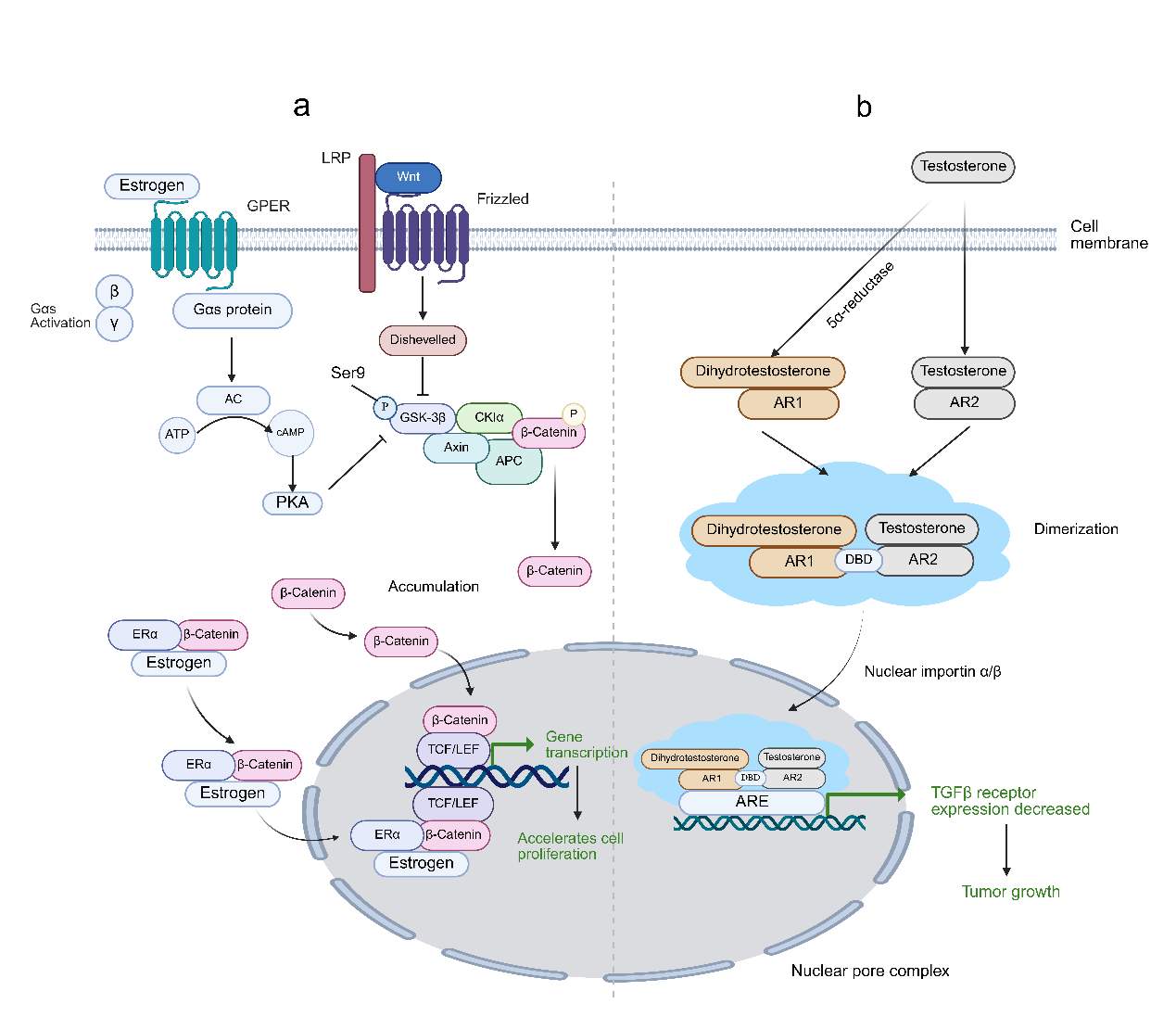


Figure 3 (a): Estrogen-binding plasma membrane G protein-coupled estrogen receptor (GPER) activates Gs protein, induces adenylyl cyclase (AC) to catalyze ATP to produce cAMP, and activates protein kinase A (PKA). PKA phosphorylates glycogen synthase kinase 3β (GSK-3β) serine (p-Ser9) at position 9, inhibits its kinase activity, blocks the function of β-catenin (β-catenin) degradation complex, and leads to the accumulation of phosphorylated β-catenin in the cytosol. After translocation of β-catenin into the nucleus, it forms a transcriptional complex with T cytokine/lymphoid enhancer (TCF/LEF). It up-regulates the expression of cyclin D1 to drive cell proliferation. At the same time, estrogen diffuses into the cell. It binds to the nuclear receptor ERα, which directly enhances the transcriptional activity of the β-catenin/TCF/LEF complex and synergistically promotes the expression of Wnt target genes.

(b):After testosterone crosses the plasma membrane, it is partially converted into dihydrotestosterone (DHT) catalyzed by 5α-reductase, which binds to the androgen receptor (AR) to form a hormone-AR complex, respectively. The two monomers are dimerized by a DNA-binding domain (DBD) and transported into the nucleus via the nuclear pore complex (NPC) mediated by the Importin α/β complex. Intranuclear AR dimers specifically bind to androgen response elements (AREs) and inhibit the transcription of transforming growth factor β receptor (TGFβR) genes, resulting in down-regulation of TGFβ receptor expression, relieving its tumor suppressor function, and indirectly promoting tumor progression. This figure is an original schematic created by the authors to summarize key mechanisms based on the cited references. It is important
